# Lipid Droplets, Perilipins and Cytokeratins – Unravelled Liaisons in Epithelium-Derived Cells

**DOI:** 10.1371/journal.pone.0063061

**Published:** 2013-05-21

**Authors:** Hans Heid, Steffen Rickelt, Ralf Zimbelmann, Stefanie Winter, Heiderose Schumacher, Yvette Dörflinger

**Affiliations:** Helmholtz Group for Cell Biology, German Cancer Research Center (DKFZ), Heidelberg, Germany; German Cancer Research Center, Germany

## Abstract

Lipid droplets (LDs) are spherical accumulations of apolar lipids and other hydrophobic substances and are generally surrounded by a thin cortical layer of specific amphiphilic proteins (APs). These APs segregate the LDs from the mostly polar components of the cytoplasm. We have studied LDs in epithelium-derived cell cultures and in particular characterized proteins from the perilipin (PLIN) gene family - in mammals consisting of the proteins *Perilipin, Adipophilin, TIP47, S3-12 and MLDP/OXPAT (PLIN 1-5)*. Using a large number of newly generated and highly specific mono- and polyclonal antibodies specific for individual APs, and using improved LD isolation methods, we have enriched and characterized APs in greater detail and purity. The majority of lipid-AP complexes could be obtained in the top layer fractions of density gradient centrifugation separations of cultured cells, but APs could also be detected in other fractions within such separations. The differently sized LD complexes were analyzed using various biochemical methods and mass spectrometry as well as immunofluorescence and electron– in particular immunoelectron-microscopy. Moreover, by immunoprecipitation, protein-protein binding assays and by immunoelectron microscopy we identified a direct linkage between LD-binding proteins and the intermediate-sized filaments (IF) cytokeratins 8 and 18 (also designated as keratins K8 and K18). Specifically, in gradient fractions of higher density supposedly containing small LDs, we received as co-precipitations cytidylyl-, palmitoyl- and cholesterol transferases and other specific enzymes involved in lipid metabolism. So far, common proteomic studies have used LDs from top layer fractions only and did not report on these transferases and other enzymes. In addition to findings of short alternating hydrophobic/hydrophilic segments within the PLIN protein family, we propose and discuss a model for the interaction of LD-coating APs with IF proteins.

## Introduction

In mammals the perilipin (PLIN) family of lipid droplet (LD)-binding proteins comprises five members: perilipin, adipophilin, TIP47, S3-12 and MLDP/OXPAT (also named PLIN1-5 [1]). It has been shown, that the absence of perilipin in mice leads to lean animals and reverses obesity [2], [3]. In recent years numerous reports have shown an involvement of LDs and specific PLIN proteins in a variety of diseases including fatty liver disease, hepatosteatosis, atherosclerosis, renal lipid disorders, giant axonal neuropathy and cancer, as well as viral infection and proliferation (e.g. caused by hepatitis C virus - HCV; see, e.g., [4]–[17]). Despite these numerous reports on LDs, relatively little is known neither about the underlying protein compositions and structures nor about the mere factors which regulate LDs in general. LDs are now recognized as not just a mere deposition pool of triglycerides, but as independent organelles of their own.

The aim of our studies was to elucidate molecular interactions between LDs and AP molecules and to identify possible architectonic LD protein associations. Working on LD-binding proteins, testing newly generated mono- and polyclonal antibodies specific for LD-associated proteins and using the epithelium-derived culture lines PLC and CaCo-2, we discovered specific interactions of LD proteins with intermediate-sized filaments (IFs). Earlier literature reports postulated close association of IFs with LDs in general (see [6], [18]–[28]). However, whether IFs were only randomly adjacent to LDs or directly tethered by unknown associated proteins was not demonstrated. These previous studies used mainly adipocytes, i.e. cells expressing IFs of the vimentin-type. Only Heid et al. [6] so far included the possibility of a cytokeratin filament-LD association and involvement in other cells, e.g. in epithelium-derived CaCo-2 cells. Among other methods, these studies used conventional electron microscopy and showed that vimentin IFs can be abundantly seen in close vicinity of LDs [23]. In addition, the accumulation and the fusion of LDs could be influenced and stopped by disrupting the vimentin IF network (shown in 3T3-L1 cells by Lieber & Evans [26]). By providing further evidence, including immunoelectron microscopy showing LD-PLIN-cytokeratin linkages we propose a model for these protein interactions.

## Materials and Methods

### Antibodies and Reagents

Primary monoclonal (mab) and polyclonal (pab) antibodies generated against human and mouse members of the PLIN family of LD-binding proteins are listed in **Table S1** (for other antibodies used in this study, see e.g., Franke and Rickelt [29]). In addition, pab specific for the intermediate-sized filaments vimentin, cytokeratins 8 and 18 as well as further mabs specific for LDL-receptor, cytokeratins 8 (clone Ks 8.7) and cytokeratins 5+8 (clone C22, pan-keratin) were commercially obtained (Progen Biotechnik, Heidelberg, Germany). Mabs specific for golgin 97 (clone CDF4; Molecular Probes, Life Technologies GmbH, Darmstadt, Germany) and calnexin (clone AF-18; Thermo Fisher Scientific, Bonn, Germany) were used for comparisons.

Secondary antibodies used were Alexa Fluor 488-coupled (MoBiTec, Göttingen, Germany) as well as the specific Cy3-coupled antibodies (Dianova, Hamburg, Germany). For immunoblot analysis, horseradish peroxidase-conjugated secondary antibodies were applied (Dianova).

Oleic acid (OA) complexed with bovine serum albumin (BSA; Sigma-Aldrich, Taufkirchen, Germany) was applied to the cell culture media (usually 100 µM/ml OA for 2–3 h; in some cases, when larger-sized LDs or greater amounts of LDs were needed, for 24 h) prior to fixing or harvesting the cells.

### Cell Culture

Cell cultures of human hepatocellular carcinoma line PLC (CRL-8024) and colon carcinoma line CaCo-2 (HTB-37) were obtained from the American Type Culture Collection (ATCC; LGC Standards GmbH, Wesel, Germany).

### Gel Electrophoresis and Immunoblotting

For biochemical experiments, cultured cells were washed three times with phosphate-buffered saline (PBS) and scraped off in sodium dodecylsulfate (SDS) sample buffer (250 mM Tris-HCl, pH 6.8, 2% SDS, 10% glycerol, and 125 mM dithiothreitol (DTT)) and homogenized. Lysates of cells were treated with benzonase (Merck, Darmstadt, Germany), boiled for 3 minutes and used for gel electrophoresis. SDS-polyacrylamide gel electrophoresis (SDS-PAGE) and immunoblotting was essentially as previously described [30], [31]. Routinely we used 4–20% minigels (Anamed Elektrophorese GmbH, Groß-Bieberau, Germany). For screening purposes, SDS-PAGE gels without sample combs (“curtain gels”) were used, proteins transferred onto polyvinyliden fluoride (PVDF) membrane and primary antibodies were applied in a multi-slot apparatus (Biometra, Goettingen, Germany).

### Nitrogen Cavitation, Density Gradient Centrifugation

For the isolation of LDs from culture cells, a modified method of Brasaemle [32] was used, using shorter OA stimulation and nitrogen cavitation for cell disruption, instead of narrow gauge needles. The self-generating gradient media OptiPrep (Iodixanol) for density gradient centrifugation (Progen) and a tube slicer apparatus (Beranek, Weinheim, Germany) to collect the floating fat layer of gradient separations were found to be essential for LD enrichment and isolation. Briefly, the medium was removed from the cells; cells were washed twice with PBS and replaced by PBS containing protease inhibitors (Roche Diagnostics GmbH, Mannheim, Germany). Cells were harvested with a rubber policeman in tubes with perforated lids and placed in a cavitation apparatus (Parr Instrument GmbH, Frankfurt, Germany). Nitrogen pressure was applied at 700–800 psi for 10 min. After pressure release, the appearing gas bubbles of the lysed cell suspension were removed by vortexing followed by brief centrifugation at 1000 *g* also in order to get rid of gross cell debris. The supernatant was directly taken in SDS sample buffer or used for gradient separation centrifugation mixed with an equal volume of gradient medium Iodixanol (Iodixanol). Depending on the amount and volumes of the lysed cell material, the resulting suspension of 30% of Iodixanol was placed in ultracentrifugation tubes (either SW40 or SW60 tubes; Beckman Coulter GmbH, Krefeld, Germany). This bottom layer was overlaid with 20% and 10% Iodixanol mixtures in PBS and finally with PBS supplemented with protease inhibitors. The quality of the gradients after centrifugation [3 h at 4°C and 40.000 rpm (SW40TI) or for 2 h at 50.000 rpm (SW60)] was controlled by measuring the refraction index of the individual fractions (Refractometer; Carl Zeiss Microscopy GmbH, Goettingen, Germany). LD layers on top of the gradient were collected with a tube slicer. The gradient fractions beneath the LD layer of the ultracentrifugation separation were collected by careful pipetting. For washes of 1 volume of LD fraction, 4 volumes of “high salt” buffer was added (1.5M NaCl, 5 mM EDTA, 5 mM EGTA in PBS plus protease inhibitors) and the mixture slowly pipetted up and down (20–30 times) using a plastic tip which by appropriate cutting had a broad opening to avoid shearing forces. The obtained milky suspension was mixed with an equal volume of OptiPrep media and separated by a second ultracentrifugation run.

Concentration of protein fractions obtained from by density gradient centrifugation and removal of hydrophobic substances was by methanol precipitation: 4 volumes of methanol were added to one volume of protein solution. After mixing, samples were stored for several hours at −20°C and final centrifugation was at 16.100 *g* for 30 min at 4°C. Supernatants were removed, the residual pellets were dried and suspended in SDS sample buffer for gel electrophoresis or in RIPA buffer for immunoprecipitations (IPs, see below).

### Immunoprecipitation

For IPs, the resulting sediments of the methanol precipitations were dissolved by vortexing in Triton X-100–containing IP buffer (RIPA buffer; 20 mM Hepes, pH 7.4, 150 mM NaCl, 5 mM EDTA or 0.5 mM CaCl_2_, 1% Triton X-100, 0.5% sodium deoxycholate, 0.1% SDS, 1 mM DTT and protease inhibitors). The supernatant obtained after centrifugation (16.100 *g* for 15 min at 4°C), was precleared with protein G– or protein A–coupled magnetic beads (Dynal Dynabeads; Invitrogen, Darmstadt, Germany) for several hours. In parallel, protein A– and/or protein G–coated magnetic beads were incubated with the appropriate antibodies or with control antibodies in buffer containing 50 mM Tris-HCl (pH 7.5) at 4°C. The precleared supernatants were incubated with the antibody-coupled beads overnight at 4°C. Beads obtained were washed several times with PBS and finally boiled in SDS sample buffer. After SDS-PAGE, gels were transferred onto PVDF membranes and used for immunoblotting or silver stained and used for mass spectrometry (MS) analysis (see below).

### Protein-protein Binding Assay

Recombinant human proteins of the PLIN family (*Adipophilin-rec* and *TIP47-rec*) were tested in different combinations with recombinant proteins of the IF family (Cytokeratin 8, *hCK8-rec*; Cytokeratin 18, *hCK18-rec;* Progen) as well as with bovine native IF proteins (*bCK8-nat, bCK18-nat; Progen)* for their ability to bind and interact. The recombinant proteins were separated by SDS-PAGE and transferred to PVDF membranes respectively. In contrast to skim milk solutions usually used for membrane blocking, membranes were blocked with 0.2% Tween 20 in PBS because milk contains *per se* high amounts of adipophilin and TIP47 which can interfere with antibody reaction. Buffers and conditions for working with recombinant and native IF proteins were essentially taken from the *in vitro* IF reconstruction experiments of Herrmann et al. [33]. Incubations of membranes with recombinant or native proteins were in 10 mM phosphate buffer pH 7.4-0.05% Triton X-100 at room temperature. Protein concentration for incubation was 1–2 µg per ml PBS. Incubation of proteins was 30 min, primary monoclonal antibody was added and the incubation continued for 30–40 min, followed by 3 washes with 20 mM phosphate buffer with 0.05% Triton X-100 and the incubation of secondary HRP-coupled antibody (45 min) and several 5 min washes with PBS without detergent, before enhanced chemiluminescence (ECL) reaction. Membranes in parallel were incubated for controls either without recombinant or native proteins, solely by primary and secondary antibodies, or alternatively without proteins and primary antibody, solely by secondary antibody.

### Mass Spectrometry

Nondestructive silver staining of SDS gels was according to the recommendation of the Protein Analysis Service at the German Cancer Research Center (DKFZ). Gel bands from protein lanes were processed by the Protein Analysis Core Facility Unit at the DKFZ and analyzed with nanoLC-electrospray-tandem mass spectrometry (nanoLC-ESI-MS/MS) and an Orbitrap system.

### Immunofluorescence Microscopy

Immunofluorescence microscopy of cultured cells was performed as previously described [31]. For normal cell culture and methanol/acetone fixation of cells no precoating of glass coverslips was necessary. In case of formaldehyde (FA) fixation, cells were grown on glass coverslips coated with 0.01% poly-L-lysine. For fixation of LDs within cells preferentially PBS containing 2% formaldehyde at RT for 10–20 min was used followed by washing three times for 5 min with PBS containing 50 mM NH_4_Cl to quench residual-free aldehyde groups. Thereafter, two permeabilization steps using PBS containing 0.2% saponin for 7–10 min at RT followed prior the application of primary antibodies. For staining of IF proteins, methanol/acetone fixation was preferred. Therefore, cells grown on glass coverslips were rinsed in PBS and fixed for 5 min in methanol followed by acetone (20 s), both pre-cooled at −20°C. The samples were then briefly air dried and rehydrated in PBS prior to the immunostaining procedure. After permeabilization with 0.2% Triton X-100 in PBS for 5 min at RT, cells were washed several times in PBS.

In general following both fixation methods, the primary antibodies were applied for 1 h at RT followed by three washes in PBS (5 min each), and incubation with the appropriate secondary antibodies (45 min at RT), again washed with PBS (3×5 min), a short rinse in distilled water, and final dehydration in 100% ethanol (1 min). After air drying, the specimens were mounted with Fluoromount G. Fluorescence was documented with a photomicroscope (Axiophot II equipped with an AxioCam HRI; Carl Zeiss, Jena, Germany). For confocal laser-scanning immunofluorescence microscopy, LSM 700 and LSM 780 microscopes (Carl Zeiss) were used.

### Electron- and Immunoelectron Microscopy

The electron microscopy protocols were essentially as described (see e.g. [23], [31], [34]). Isolated LD fractions were fixed with 2.5% glutaraldehyde in 50 mM sodium cacodylate buffer (pH7.2, containing 50 mM KCl and 2.5 mM MgCl_2_) for 20–30 min, washed three times in the same buffer, postfixed in 50 mM cacodylate buffer containing 2% OsO_4_ for 2 h, and repeatedly rinsed in this buffer. All steps were at 4°C. Thereafter, the tissue blocks were stained overnight with 0.5% uranyl acetate in water (“aqua iniectabilia”; B.Braun, Melsungen, Germany) washed, dehydrated, and embedded in Epon. In some cases simultaneous glutaraldehyde-osmium tetroxide fixation was used (see [35]).

Immunoelectron microscopy was performed as described (see, e.g. [29], [31]). Ultrathin sections were prepared with a Reichert-Jung ultramicrotome (Leica Microsystems, Wetzlar, Germany). Electron micrographs were taken at 80 kV using an electron microscope (EM 900; Carl Zeiss).

## Results

**Figure 1:**
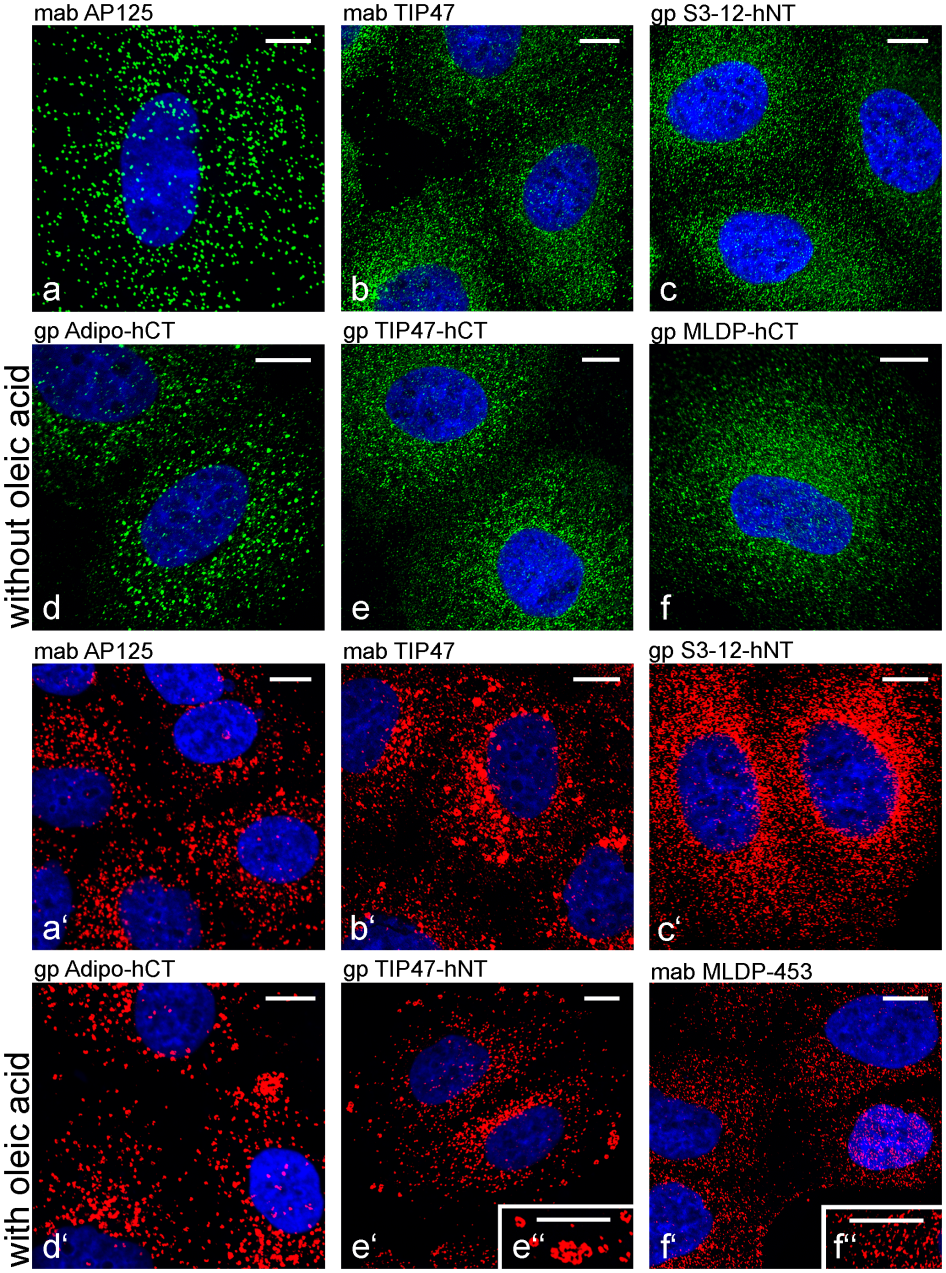
Laser scanning immunofluorescence microscopy of lipid droplets (LDs) in cultured hepatocellular carcinoma cell line PLC. (a–f; green) Normal cells after 2% formaldehyde fixation and incubation with various newly generated antibodies against PLIN proteins. (a′–f′; red) Oleic acid (OA; 3 h) stimulated cells. Numerous LDs could be detected by monoclonal antibodies (mabs) AP125 (adipophilin; a,a′), TIP47.49.19 (TIP47; b,b′), MLDP-453 (MLDP; f′) and polyclonal antibodies (pabs) Adipo-hCT (adipophilin; d,d′), TIP47-hCT (TIP47; e), TIP47-hNT (TIP47; e′), S3-12-hNT (S3-12; c, c′), MLDP-hCT (MLDP; f). Note: Distribution and sizes of LDs in individual cells might differ slightly in normal cells, depending on the growth phase, cell division status or addition of fresh culture media. (a,a′,d,d′) Adipophilin antibodies stained larger LDs while other PLIN antibodies, especially when compared to the S3-12 staining (c), detected numerous tiny LDs. Note in addition: Short OA treatment changed adipophilin and TIP47 staining in many cells (a,a′,b,b′,d,d′,e,e′). Staining appearance switched in many cells towards larger spots and ring-like structures. In contrast S3-12 and MLDP staining was not visibly influenced by OA treatment (c,c′,f,f′). Differences in sizes seen with TIP47 and MLDP staining upon OA treatment are highlighted by inserts (e′′, f′′). These data indicate that differently reacting LD populations might exist within individual cells. DAPI (blue) was used to stain the nuclei. Bars: 10 µm.

**Figure 2:**
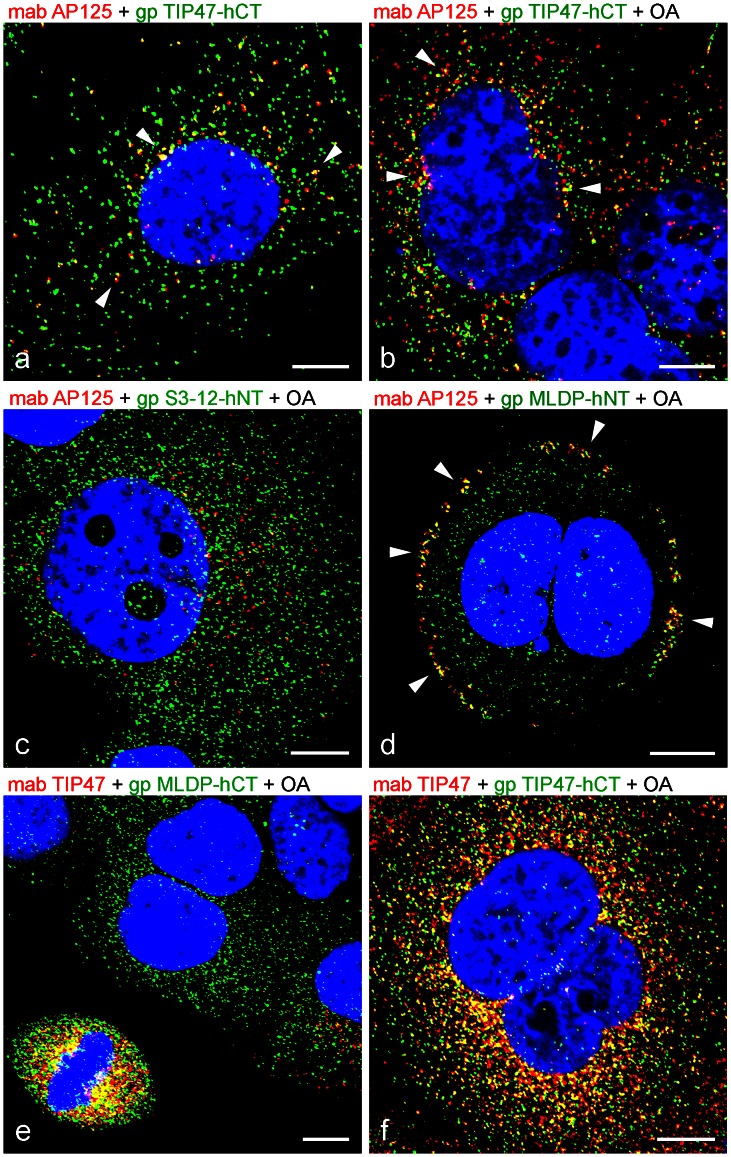
Laser scanning double-label immunofluorescence microscopy showing LD labelling comparisons of PLIN protein antibodies in PLC cells. (a) Normal, non-stimulated cells. (b–f) OA treated cells (2 h). Fixation of cells was with formaldehyde and the combination of mixtures of specific antibodies is given above the individual picture respectively. The following comparisons are shown: (a,b) Adipophilin (red) vs. TIP47 (green); (c) Adipophilin (red) vs. S3-12 (green); (d) Adipophilin (red) vs. MLDP (green); (e) TIP47 (red) vs. MLDP (green); (f) TIP47 (red) vs. TIP47 (green). Note: Whereas in non-treated cells adipophilin and TIP47 show some co-localizations in the perinuclear region (yellow mixed color; examples shown by arrowheads in a), the numbers of LDs of co-localizations is remarkably increased with stimulation (examples shown by arrowheads in b). (c) Despite of the many LDs visible, adipophilin and S3-12 are rarely found to co-localize. (d) After cell division, adipophilin and MLDP are seen with many co-localizations at the periphery of the plasma membrane (examples shown by arrowheads). (e) LDs for TIP47 and MLDP are not seen co-stained, but in cell division (lower left corner), plenty LDs densely packed with possible connections of these PLIN proteins show up. (f) At the cell periphery the two different antibodies specific for TIP47 are not always staining the same LDs. Certain LD subpopulations are recognized by one TIP47 antibody only. Nuclear staining was with DAPI (blue). Bars: 10 µm.

For this study, we raised and characterized extensively mono- and polyclonal antibodies (mabs, pabs) specific for all LD-binding proteins of the PLIN family. We used human and mouse peptides against N-terminal and C-terminal parts of proteins, as well as recombinant proteins for immunization (designation of antibodies and antigen information are listed in **Table S1**). Besides normal culture conditions, the cells used for antibody characterization were stimulated for LD production by addition of oleic acid (OA) to the culture medium. Short stimulation of 2–3 hours turned out to be sufficient to increase numbers and sizes of LDs in most cells as seen with antibodies specific for adipophilin and TIP47 and to a lesser degree, with S3-12 and MLDP (**Fig. 1**). In addition, we tested our antibodies in various combinations and with various cell stimulation, fixation and permeabilization protocols by double-immunofluorescence microscopy (examples shown in **Fig. 2**). We saw, by short OA treatment and with molecular defined samples, that myriads of tiny, small LDs could be visualized. Small LDs of normal, non-stimulated PLC cells positive for TIP47 and for adipophilin are demonstrated in **Figure 2a**. Most of these LD-binding proteins were found to be spatially separated. Some co-localizations were also visible, preferentially in the perinuclear region (yellow mixed color; marked by arrowheads). Upon OA addition to the culture cell media, positive adipophilin staining was rapidly seen together with the appearance of numerous, new LDs. Often these droplets were associated with TIP47. Obvious co-localizations of both PLIN proteins could be seen in many situations, preferentially in the perinuclear area (arrowheads in **Fig. 2b**). S3-12 antibodies revealed many, very small LDs and here hardly any co-localization could be detected with adipophilin (**Fig. 2c**). Adipophilin seemed to be involved mainly in the uptake of OA at the plasma membrane of cells in conjunction with MLDP (possible co-localizations marked by arrowheads in **Fig. 2d**)**.** In contrast to adipophilin in the example shown, MLDP stained also LDs distributed throughout the cytoplasm of the cells. TIP47 antibodies recognized and stained unique small droplets when compared with MLDP antibodies reaction (**Fig. 2e**). During cell division these proteins appeared to be packed closely together (left lower corner in **Fig. 2e**). A notable observation was made when we used and compared two different antibodies with different epitopes directed against one and the same PLIN protein. The merged staining picture of both TIP47 antibodies revealed clearly that many LDs co-localize (indicated by yellow, mixed color; **Fig. 2f**). Some LDs were recognized either solely by the monoclonal or solely by the polyclonal antibody. This different staining for one single LD-binding protein was observed mainly at the cell periphery.

These introductory experiments indicated that within cells and with short “hydrophobic” stimulation, many differently-sized and many different protein surface-covered LDs could be triggered and detected. A plethora of different types of LDs appeared within such treated cells. Consequently, we attempted to use our set of newly generated antibodies for the characterization of these LDs and their LD-binding proteins.

### Isolation of LDs from Density Gradient Top Layer Fractions

**Figure 3:**
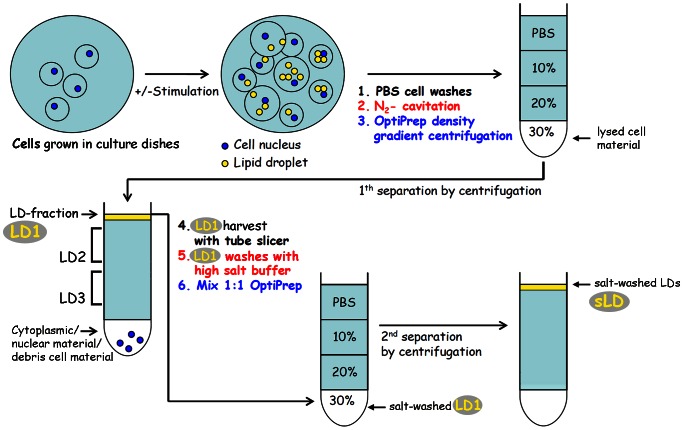
Scheme of an improved isolation and purification procedure for LDs used in the present study. Cultured cells were lysed by disintegration using nitrogen cavitation. Cell material obtained was subjected to density gradient centrifugation separation. Top layer LD1 fraction was harvested with a tube slicer. After several washing steps, including high salt washes, the LD1 fraction was subjected to a 2^nd^ gradient separation resulting in the salt-washed top layer sLD. In further experiments the combined fractions of higher densities, LD2 and LD3, as well as LD1, were used for immunoprecipitations.

We attempted to optimize the commonly used isolation procedures for LDs [32], [36]: We tried to avoid increased mechanical force (douncing, sonication, treatment with syringe needles, etc.) for cell lysis in order to get an overall more homogenous lysis and to avoid contaminations, e.g. sticking of fatty material or of viscous DNA due to disruption of the cell nucleus. Cultured cells were used as starting material for the isolation procedure and the nitrogen cavitation method was applied in the presence of protease inhibitors. With this method almost all cells could be lysed in a homogeneous manner. The lysed cell material was subjected to density gradient centrifugation immediately after lysis. The lipid and protein material within the gradient fractions was collected (see scheme in **Fig. 3**). In addition, we performed washings of the top layer LD-fraction (LD1) with “high salt” phosphate buffer in order to remove loosely bound material followed by a second gradient centrifugation, yielding salt-washed LDs (“sLDs”; cp. **Fig. 3**).

**Figure 4:**
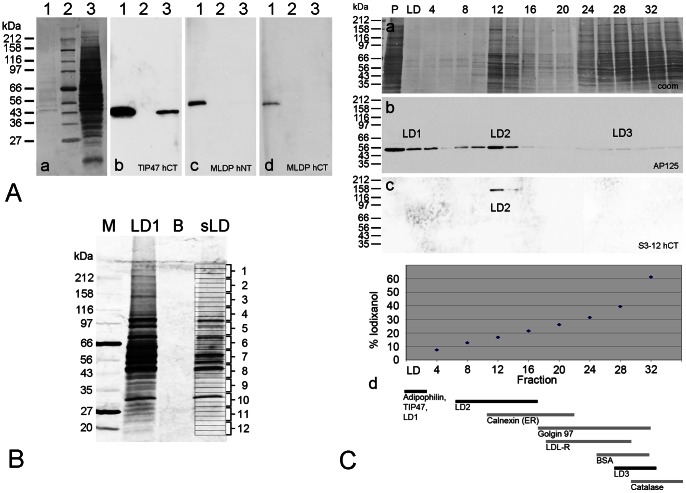
Characterization of PLIN antibodies and separation of LDs. Fig. 4A: Immunoblot characterization of antibodies specific for PLIN proteins. Instead of 3 h, PLC cells were treated for 24 h with OA in order to increase the amount of LDs. Pabs against TIP47 and MLDP were used to demonstrate the specificity of the antibodies. (a) Proteins separated by SDS-PAGE and transferred to a PVDF membrane were stained with Coomassie Blue. (b–d) Immunoreactions obtained using antibodies to TIP47-hCT (b), MLDP-hNT (c) and MLDP-hCT (d). Lane 1: Proteins from top layer LD fraction of density gradient separation (cp. LD1 in Fig. 3). Lane 2: Protein markers; on the left margin the molecular weights are given. Lane 3: Whole cell lysate. Note: TIP47 could be detected in whole cell lysate and enriched in the LD fraction (b), MLDP was detected at 52 kD by two different antibodies only after LD enrichment (c,d). Fig. 4B: Density gradient separated top layer fraction LD used for mass spectrometry analysis. LD proteins were isolated from PLC cells according to the scheme shown in Fig. 3, separated by SDS-PAGE which then was silver-stained. Position of molecular weight markers are given on the left margin. M: marker protein lane; LD1: LD fraction from 1^st^ separation; B: sample buffer lane (control); sLD: salt-washed LD fraction from 2^nd^ separation. The complete lane of sLD was cut into distinct bands, as indicated by the numbers (1–12) given at the right side and subjected to proteomic analysis. An elaborated list of results of identified proteins is displayed in Fig. S2. Fig. 4C: LD-binding proteins detected in density gradient centrifugation fractions of OA-treated PLC cells. Fractions obtained by cell disintegration using nitrogen cavitation, Iodixanol gradient centrifugation and SDS-PAGE separations are shown. Coomassie blue (Coom) stained fractions (a) were tested with adipophilin AP125 (b) and with S3-12-hCT (c) antibodies. A summary of gradient separations, showing different LD-enriched areas and positions of several proteins analyzed, is given in (d). BSA and catalase were applied in parallel gradient separations as control and detected by Coomassie staining. The positions of calnexin, golgin 97 and LDL-Receptor were obtained by Western blotting respectively. Note: Adipophilin (TIP47 similar; not shown here) was detected in three LD gradient areas (LD1, LD2 and LD3) including the top layer LD1. S3-12 could be detected only in a higher density layer (LD2).

Representative immunoblot analysis of these separations are shown in **Figure 4A**. As an example, the detection of TIP47 in whole cell lysates and the enrichment in density gradient LD1-fractions of OA treated cells is shown (**Fig. 4A** **–b**). In contrast, MLDP could not be detected readily using whole cell lysates, but it was detectable in LD1-fractions obtained from cells only after longer OA treatment (24 hours instead of normally used 2–3 hours for increased amounts of LDs; **Figs. 4A** **–c, 4A–d**). Additional examples of immunoblots for the characterization of the new antibodies are given in (**Fig. S1**).

To identify the proteins present in such purified, salt-washed LD1 fractions, a complete silver stained gel lane (“sLD” shown in **Fig. 4B**) was subjected to mass spectrometry (MS) analysis. More than 650 proteins could be identified (for extended list of results obtained with fraction sLD and detailed information, assignments and accession numbers, see **Fig. S2**). The general result is briefly summarized in the following: **(1)** LD-binding proteins known from the literature could be detected – often with very high scores, including adipophilin, TIP47, AUP1 homolog protein, hypoxia up-regulated protein 1, 100 kDa coactivator protein, ABHD5 (CGI-58), rab-18. **(2)** Many proteins involved in fatty acid- and lipid-pathways were found. **(3)** Many annotated endoplasmic reticulum (ER) proteins could be identified. **(4)** Many cytoskeletal and junctional proteins, including cytokeratins 8 and 18 with high scores, were identified. **(5)** Many receptor proteins, transmembrane proteins, ion channel proteins, ATPases, GTPases, Kinases, Ras- and Rab-family members were detected. **(6)** Relatively few proteins typical for Golgi apparatus, mitochondria, peroxisomes, nucleus, endosomes and lysosomes could be identified.

In addition, the PLIN proteins adipophilin and TIP47– besides appearance in SDS gel with normal molecular weights of 53 and 50 kD respectively– could be found also within unusual high molecular weight areas.

To control the purity of the obtained density gradient fractions in more detail, we analyzed the top layers fractions, LD1 and sLD, by “simultaneous fixation” and electron microscopy (EM, for methodology see [35]; **Fig. S3**). Our isolation procedures - including salt-washed LDs - did not yield pure LDs. Besides the many LDs with an average size of 1–2 µm diameters, which were often seen packed inside bag-like clusters, we found many LD-unrelated contaminants, cytoplasmic inclusions, membranous fragments, parts of other cell organelles and other undefined, electron-dense material. In the top layer gradient fractions, presumably we encountered fused, big LDs together with unspecific sticking cell material. These impurities might give an explanation for the diverse scores in mass analysis not related to lipid metabolism.

### LDs in Gradient Fractions of Higher Densities

After having examined the gradient top layer LDs, we turned to small and tiny LDs, and we searched for the identification of small LDs associated proteins. Small LDs are *per se* not expected to ascent to the top of the centrifugation gradient because, at very early stages in LD biogenesis, the amount of their lipid content is thought to be rather low. Supposedly these LDs would be found enriched in gradient fractions with higher density. Therefore we analyzed the complete gradient separations by immunoblot analysis. An example of such experiments is given in **Figure 4C**. LD-associated proteins could be detected within the top layer of the gradients (LD1), but - mainly due to the short time of stimulation - also in other fractions (LD2, LD3). For example, adipophilin could be found essentially in three different fraction areas: **1)** at 0–5% iodixanol concentration of the gradient (top layer, LD1); **2)** at 5–15% (LD2); **3)** at 15–25% (LD3) (see **Fig. 4C** **–a,b**). In contrast S3-12 could be detected only at higher density of 5–15% iodixanol (LD2) and not within the top layer (**Fig. 4C** **–c**).

**Figure 5:**
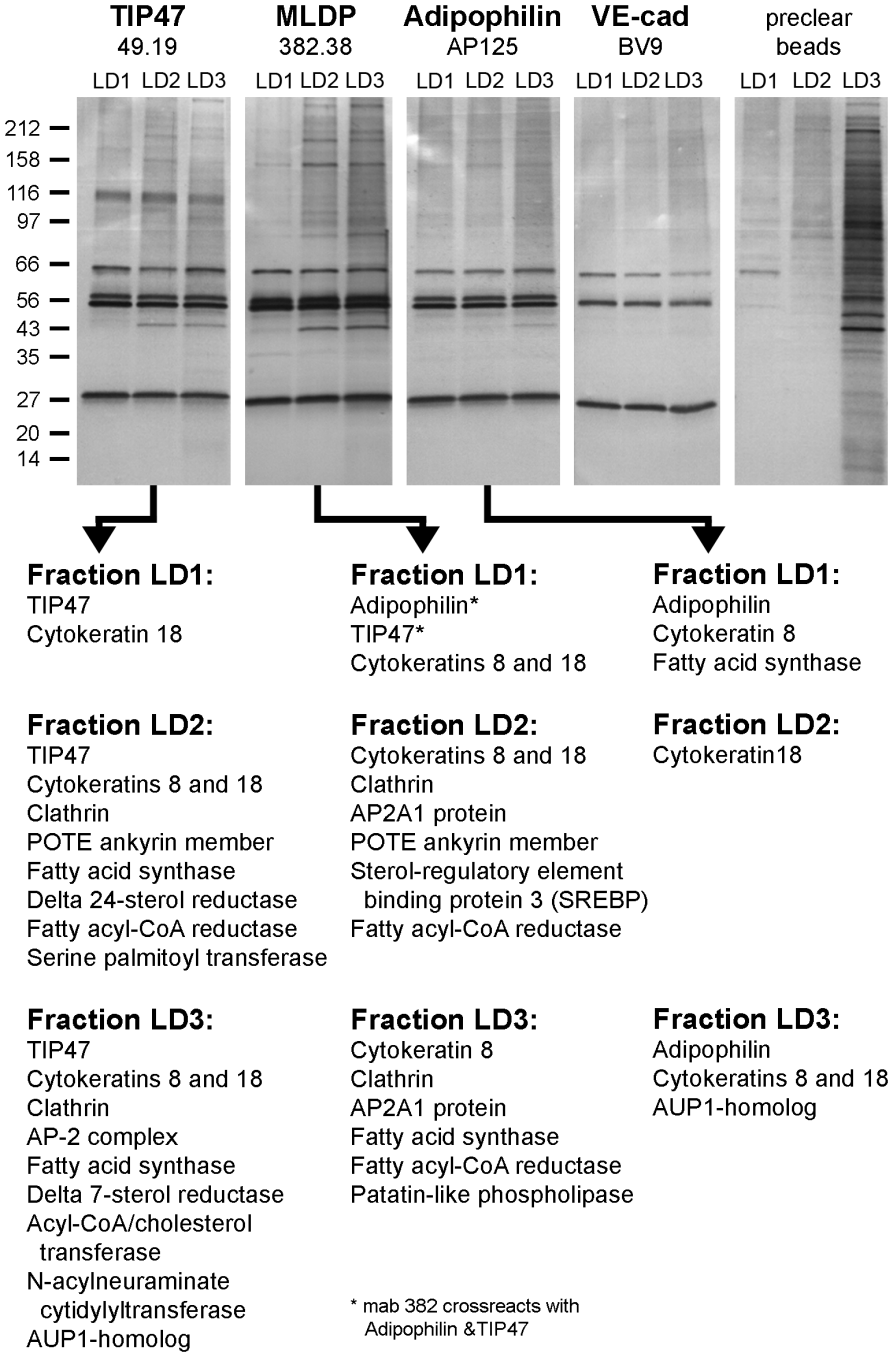
Summarized results of immunoprecipitations (IPs) using OA treated PLC cells and PLIN antibodies followed by MS analysis. Proteins from gradient separations as seen in scheme of Fig. 3 and in Fig. 4C of the top layer LD fraction (LD1; 0–5% iodixanol), combined fractions LD2 (5–15% iodixanol) and combined fractions LD3 (15–25% iodixanol) were used for precipitation. IPs obtained with mabs TIP47, MLDP-382, AP125 and as a control VE-cadherin were analysed by SDS-PAGE and silver staining respectively. Positions of molecular weight marker proteins are given on the left margin. For MS analysis visible bands from fractions, differing from bands seen in unbound lysate supernatant material as well as from control antibody, were chosen (for annotation of individual gel bands used for MS analysis and for detailed lists of identified proteins see Fig. S4. A survey of major MS results obtained is given in the lower part of the figure. Note, besides members of the PLIN family, especially cytokeratins 8 and 18 could be precipitated. In addition, specific enzymes and proteins involved in lipid metabolism and endocytosis could be detected - predominantly seen with TIP47 antibodies and in fractions of higher densities, including sterol reductases, fatty acyl-CoA reductase, acyl-CoA/cholesterol transferase, serine palmitoyl transferase and N-acylneuraminate cytidylyltransferase.

Therefore we included gradient fractions with higher densities in our immunoprecipitation (IP) experiments. A summary of results obtained with such IPs combined with MS analyses is shown in **Figure 5**. We used four monoclonal antibodies (mabs) - specific for TIP47, for adipophilin, for MLDP and one control antibody – to analyze gradient fractions of OA stimulated PLC cell material. Within all three collected gradient fractions used (LD1, LD2 and LD3), the LD-binding proteins TIP47 and/or adipophilin could be immunoprecipitated. As the most striking result, we obtained cytokeratins 8 and 18 as co-precipitation products with high scores upon mass spectrometry. These cytokeratins were not precipitated with the control antibody. Other co-precipitated proteins received are known to be involved in lipid binding and lipid metabolism. As a further most intriguing result in fractions of less buoyancy, we could co-precipitate with the TIP47 antibody cytidylyl-, palmitoyl- and cholesterol transferases and other enzymes involved in lipid metabolism. In addition with mabs TIP47 and MLDP (used mab MLDP382.38 cross-reacts with TIP47 and adipophilin) we were able to pull-down, delta-7-sterol reductases, delta-24-sterol reductases, sterol-regulatory element binding protein 3 (SREBP-3) and POTE ankyrin member. Also co-precipitated were fatty acyl-CoA reductases needed for the conversion of fatty acids to fatty alcohols and required for the synthesis of ether lipids. The detected patatin-like phospholipase cleaves fatty acids from membrane lipids and was found by MLDP antibody precipitation in a high density gradient fraction. In addition all three mabs were able to precipitate fatty acid synthase (FAS). Using adipophilin antibodies FAS was also detected in the top layer LD1 fraction. The LD-associated protein AUP1-homolog was precipitated with mabs specific for TIP47 and adipophilin and found in fraction LD3. Clathrin and AP2 proteins involved in endocytosis could be tagged with mabs specific for TIP47 and MLDP in fractions LD2 and LD3, but not within the top layer LD1 (**Fig. 5**; for more details see **Fig. S4** and Discussion).

### PLIN-protein and Intermediate Filaments Alliances

**Figure 6:**
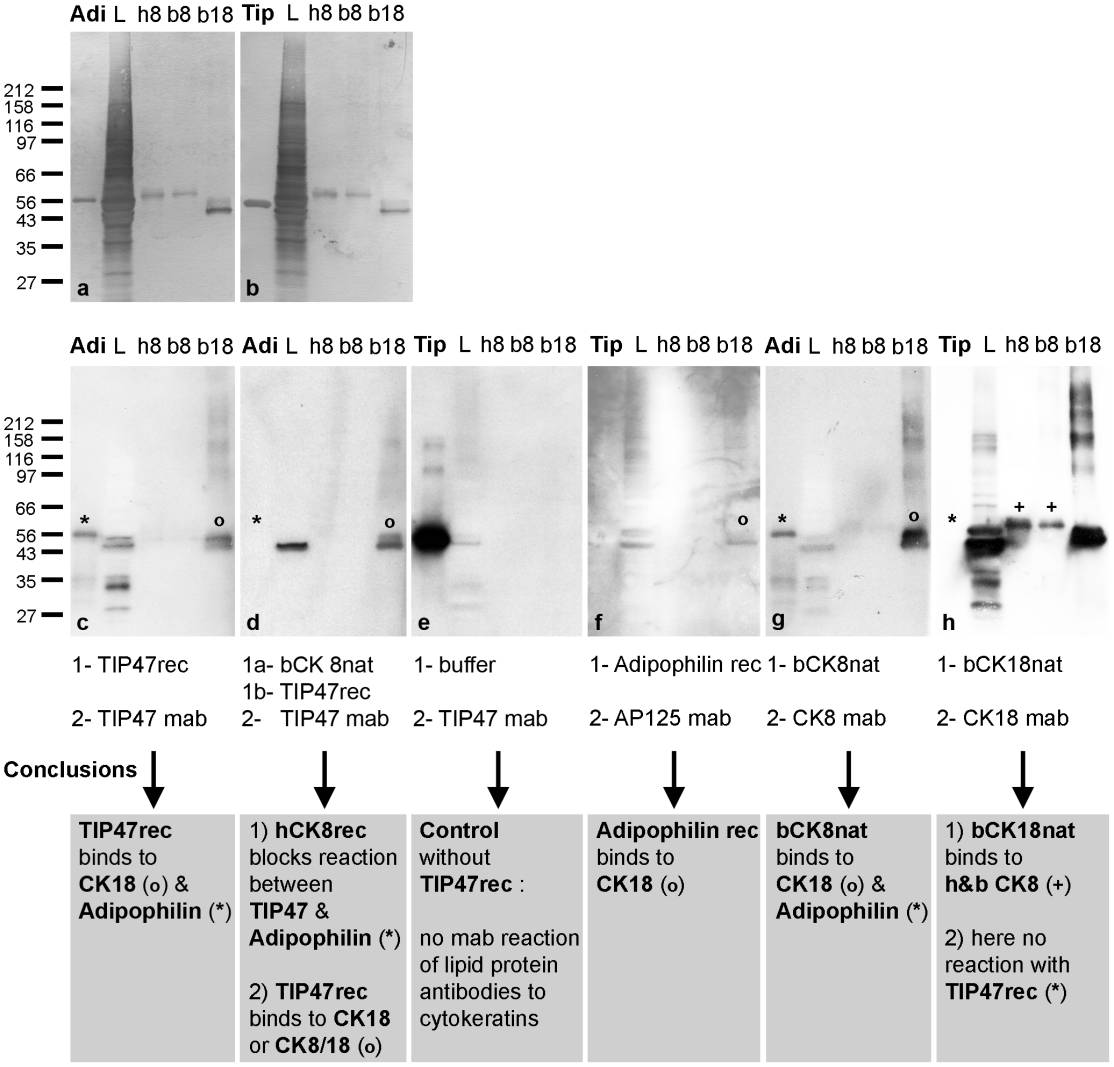
Protein-protein binding assays using recombinant (rec) and native (nat) cytokeratins and recombinant PLIN proteins. Various PLIN and IF proteins were separated by SDS-PAGE, transferred to PVDF membranes and used for testing. Examples of Coomassie blue stained membranes used for probing are shown in a, b. Individual WB reactions (c–h) and applied incubation schedules (written below the respective membranes) are given. AP = Adipophilin rec; Tip = TIP47rec; L = whole cell lysate of PLC cells; h8 = human Cytokeratin 8 rec; b8 = bovine Cytokeratin 8 nat; b18 = bovine Cytokeratin 18 nat. Molecular weight marker proteins are given on the left margin. Specific reactions are highlighted by “*”, “o” and “+”. Summarized conclusions of blotting results are given in grey boxes below. Note, certain assay combinations suggest specific and direct protein-protein binding of PLIN proteins with cytokeratins. Only certain sandwich combinations show positive binding results. For example, TIP47 binds to PVDF-bound cytokeratin 18 (c), however, cytokeratin 18 does not react with PVDF-bound TIP47 (h). Adipophilin was able to bind to PVDF-bound cytokeratin 18 (f) and cytokeratin 8 can bind to PVDF-bound adipophilin (g). In addition, linkage between TIP47 with PVDF-bound adipophilin can be identified (c) but *vice versa* this binding cannot be detected between adipophilin with PVDF-bound TIP47 (f). The expected binding between the partners of the type I and type II intermediate filaments, i.e. of cytokeratin 8 and cytokeratin 18, is seen in d,g,h.

In order to confirm the IP results of identified cytokeratins 8 and 18, we tested in various combinations the capacity of purified native and recombinant IF proteins to interact with PLIN proteins. Examples of such binding assays (“Far Western blots”) are presented in **Figure 6**. Overall, we found indeed several combinations which demonstrated that adipophilin, as well as TIP47, could bind cytokeratins and vice versa. Distinct combinations of protein binding assays showed positive PLIN-IF binding results. For example, TIP47 was found to bind to PVDF-bound cytokeratin 18 (**Fig. 6** **c,d**). In contrast, cytokeratin 18 does not react with PVDF-bound TIP47 (**Fig. 6h**). On the other hand, buffer conditions and the binding on PVDF of a given individual protein might not be optimized yet and folding and accessibility of domains responsible for PLIN-IF protein-protein bindings might be blocked in these “in vitro” experiments. Adipophilin did bind to cytokeratin 18 (**Fig. 6f**) and cytokeratin 8 to adipophilin (**Fig. 6g**). In addition, we also noted signs of a direct adipophilin-TIP47 protein interaction (see **Fig. 6c**).

**Figure 7:**
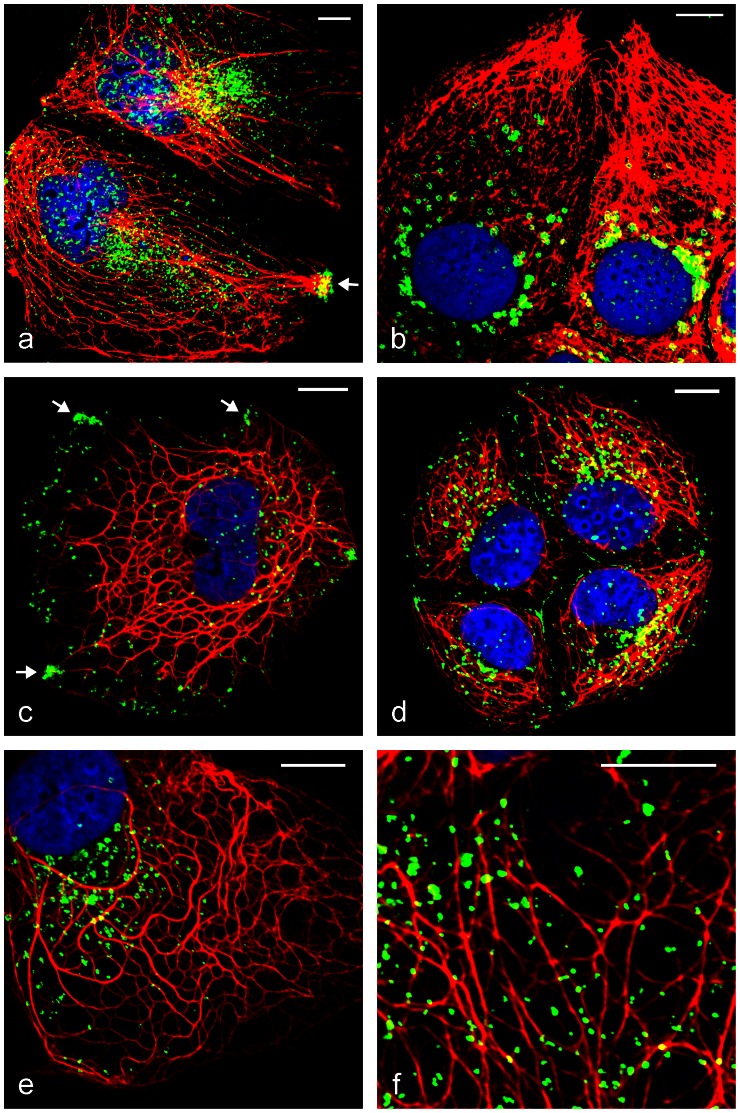
Laser-scanning double-label immunofluorescence microscopy of adipophilin and cytokeratins in OA stimulated cells showing co-localization. (a,c–f) PLC cells and (b) CaCo-2 cells after methanol fixation and incubation of primary antibodies mab C22 pan-cytokeratin (a–f; red), pab Adipo-hNT (a–c,e,f; green) and pab Adipo-hCT (d; green). Note: local accumulations of adipophilin (arrows) close to the cell plasma membrane in (a) and (c) possibly indicating sites of OA uptake. CaCo-2 cells, compared to PLC cells, form generally more dense IF networks with fewer, bigger LDs accumulated in the perinuclear region (cp. a, b). Several droplets could be seen directly co-localized to IFs (yellow, mixed color in e and f). Note in addition, faint plasma membrane staining in b,d. Nuclear staining was with DAPI (blue). Bars: 10 µm.

**Figure 8:**
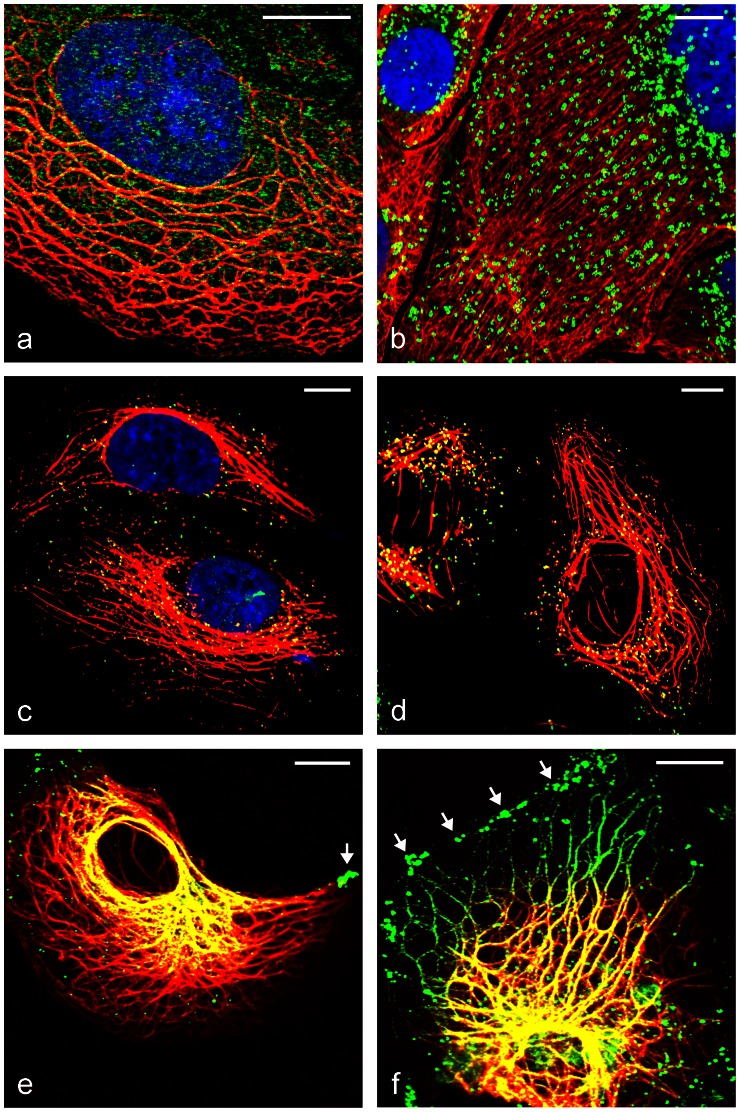
Laser-scanning double-label immunofluorescence microscopy of TIP47 and cytokeratins in OA stimulated cells showing co-localization. (a,c–f) PLC and (b) CaCo-2 cells was either fixed with methanol (a,b) or with formaldehyde (c–f) prior to incubation with primary antibodies mab C22 pan-cytokeratin (a,b,e,f; red), pab CK8 (c,d; red), pab TIP47-hNT (a,b,e,f; green) and mab TIP47.49.19 (c,d; green). Note: co-localization seen as mixed yellow color. Uptake of OA by accumulation of LDs at the cell periphery is marked by arrows (e,f). Note: By examining many of such staining’s we speculate that the droplets were transported in cells alongside IFs towards the perinuclear area. For further examples of TIP47 staining see Fig. S5. Nuclei stained with DAPI (blue). Bars: 10 µm.

We performed and revisited several double-immunofluorescence microscopy experiments, using different fixation methods and looked for co-localization and possible interactions of these two protein groups (**Figs. 7** **,** **8**). Thereby we observed some “holes” in otherwise orderly arranged IF bundles and especially there specific LD staining’s were visible (e.g. in **Figs. 7b** **,** **8b**) pointing to a close relationship of IFs and LDs. Moreover, special IF arrangements and IF bundles were seen at the cell periphery near LD accumulations, the latter presumably caused by OA uptake (**Figs. 7a,c** **; 8e,f**). Many presumable PLIN-IF co-localizations could be seen at higher magnification as yellow-colored (mix of red/green staining) LDs (**Figs. 7e,f** **; 8a,c,d**). Anti-TIP47 stained LDs at the inner cell periphery and these LDs seemed to be arranged via linear arrays along a filament system accumulating in perinuclear positions (**Figs. 8e,f**). In a series of images, detailed examples of apparent associations of small LDs, positively stained for TIP47, together with IF-bundles are shown (**Fig. S5**).

**Figure 9:**
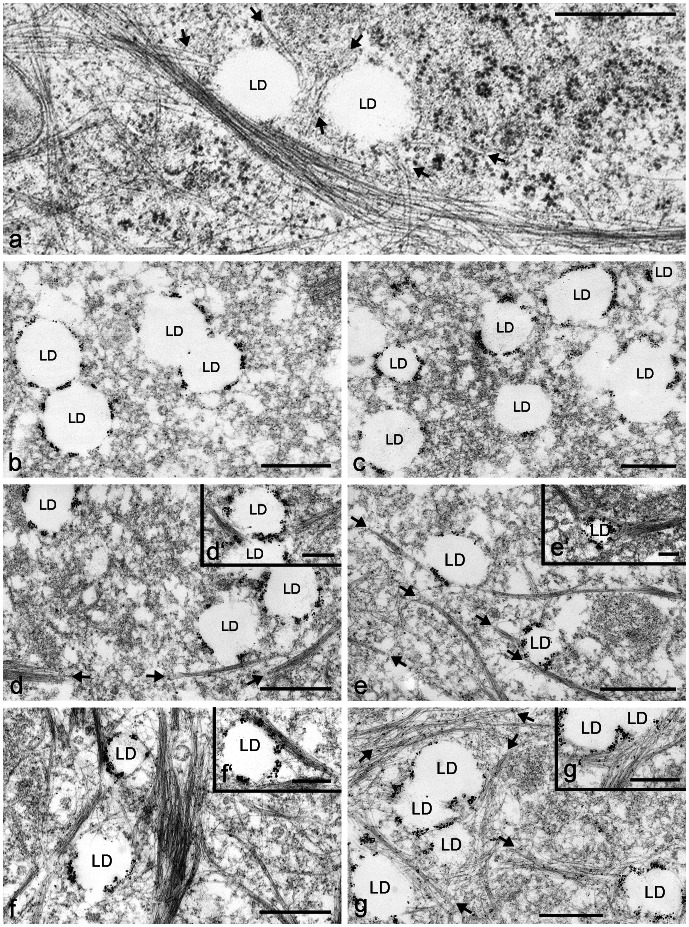
Electron microscopy and immunoelectron microscopic localization of adipophilin in OA stimulated PLC cells, revealing the direct neighborhood of LDs with IFs. (a) Bundles of intermediate-sized filaments and single filaments can be seen closely and directly associated to LDs in normal electron microscopy. (b–g) Groups of adipophilin positive LDs within the cells can be seen after nano-gold-label and silver enhancement. Examples of LD staining for adipophilin closely associated, many directly anchored, with IF bundles are shown in c-f and the inserts respectively. Several IF bundles are marked by arrows. Bars: 0.50 µm; bars in inserts: 0.25 µm.

As additional and major proof of LD–perilipin protein–intermediate filament associations (LD-PLIN-IF), we could demonstrate high resolution electron and immunoelectron microscopic localizations (EM, IEM; **Fig. 9**). Small LDs (approximately 200–500 nm in diameters) were decorated with PLIN-specific antibodies and were often seen in connection with bundles of IFs. In contrast to several recent observations by other authors, our IEM experiments, however, did not reveal tubulin in close vicinity to LDs. Furthermore, neither mitochondria nor peroxisomes could be detected directly linked to LDs.

**Figure 10:**
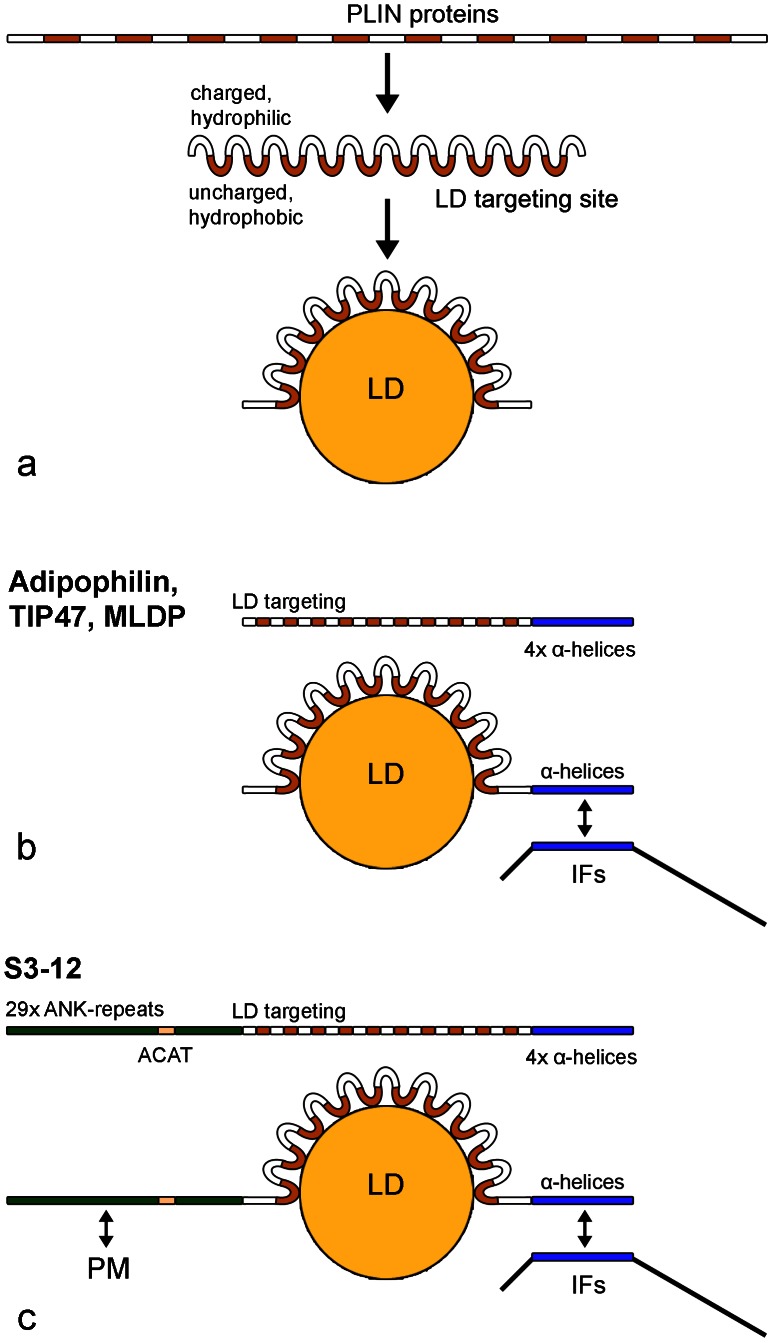
Summary of a general model for the binding of PLIN proteins to LDs and models for LD-PLIN-IF interaction. Hydrophobicity analysis revealed short alternating hydrophilic/hydrophobic stretches of amino acids all along the complete protein sequences (see **Fig. S7**). Appropriate folding can lead to a charged hydrophilic site and an uncharged hydrophobic site of proteins giving rise to a general LD targeting site. According to this model the hydrophobic site is thought to be responsible for the binding to surfaces of LDs (**a**). Adipophilin, TIP47 and MLDP possess these general LD targeting regions and additional four α-helices at the C-terminus (for α-helices of TIP47, see X-ray crystal structure data of Hickenbottom et al. [65]). Schematic drawing of protein domains are shown in the upper part of (**b**). The possible interaction and binding of α-helices of PLIN proteins with α-helices of IF proteins is schematically presented in the lower part of (**b**). S3-12 revealed 29 ankyrin-like repeats, in addition a potential acetyl-Coenzyme A acetyltransferase (ACAT) domain, a general LD targeting region and four α-helices at the C-terminus (upper part of **c**; see **Figs. S8, S9**). Potential interactions with plasma membrane (PM) via the ankyrin-like repeats of S3-12 and with IF proteins via α-helices is schematically presented in the lower part of (**c**).

We analyzed the PLIN protein sequences using various programs of the Swiss Institute of Bioinformatics (SIB) and detected alternating short hydrophobic/hydrophilic sequence patterns and, in addition for S3-12, ankyrin-like repeat units including one possible ACAT sequence motif (see **Figs. S7, S8, and S9**). As a summary of these data base findings and by combining our experimental data, we would like to present a model on possible LD–PLIN bindings and a general model for LD–PLIN–IF interactions (see **Fig. 10** and Discussion).

## Discussion

With the use of newly generated antibodies the pattern of lipid droplet (LD) staining upon oleic acid (OA) treatment of cultured cells could be investigated in detail. Many newly appearing LDs, of increasing droplet size and LD clustering were seen in epithelium-derived PLC and CaCo-2 cells upon such treatment. A switch from small droplets to larger and finally bigger droplets with ring-like, surface-stained structures could be studied using adipophilin and TIP47 antibodies. In contrast, when antibodies against other PLIN family members, S3-12 and MLDP, were used, staining was obviously not influenced as much by short time OA application (**Fig. 1**). A huge variety in expression of PLIN proteins and of different LDs was found with double immunofluorescence microscopy experiments (**Fig. 2**). These results demonstrated that cells are packed with many tiny LDs and these could be visualized by combining specific fixation methods and defined antibody staining. With commonly used neutral dyes as staining for LDs such high numbers of LDs are hardly detectable, because tiny LDs have only minor amounts of lipid content. BODIPY and Nile Red preferentially stain undefined hydrophobic contents of mainly medium-sized and large LDs. Our antibodies marked defined LD surface proteins of almost all LD sizes. BODIPY, for example, did not stain all LDs which were positively stained with adipophilin antibodies (see e.g. [37]). With LD staining of tissue sections, especially in cases of microvesicular steatosis, we recently demonstrated the advantages of adipophilin immunostaining when compared to H&E, PAS and neutral stains (see [17]).

Our immunofluorescence microscopy data revealed many different types of LDs within individual cells and allowed for detection of very small LDs upon short OA stimulation. Our experiments also showed that individual PLIN proteins seemed to show preferences for binding of certain hydrophobic cargos. Certain combinations of PLIN members seemed more suitable than single PLIN proteins to surround LD and to protect the LD contents against degradation by lipases. Amphiphilic proteins, like the PLIN proteins - which can bind to the surface of potential aggressive hydrophobic substances - are important protectors and help maintaining the integrity of biomembranes. Our findings on the variety of LD staining is in line with data recently published by Hsieh et al. [38] who showed that individual members of the PLIN protein family can sequester different LDs, some LDs associating with triacylglycerides (Plin 1a/1b – perilipin 1a/1b; Plin 5– MLDP) and others with cholesterol esters (Plin 1c – perilipin 1c; Plin 4- S3-12).

### Analysis of LDs of Top Layer Gradient Fractions

Starting with conventional proteomic analysis, we used protein material obtained from density gradient centrifugation separation and took LD fractions from the salt-washed top layer sLD. We expected that loosely bound LD contaminations could be removed by salt-washing. But we were still confronted with more than 650 identified proteins (see **Fig. S2**). Such a result is probably due to the improvements of mass spectrometry in recent years and also due to the advantages of the applied gradient medium Iodixanol. Compared to other media (e.g. sucrose or ficoll), this medium self-generates continuous gradients during centrifugation. The resulting individual zone of a sample is restricted to very narrow density fractions (see [39], ). With OA stimulated PLC cells we identified many proteins described and known for their association with LDs, in addition to proteins involved in lipid metabolism. However, we also found several unknown, or not completely described proteins which are so far not proteins related to lipid metabolism. We could verify only to a very limited extend published proteomic studies on LDs found peroxisomal, mitochondrial and nuclear proteins. Within our proteomic study, we identified adipophilin and TIP47 in gel bands around 50 kD, but also in higher molecular weight gel regions, suggesting the possibility of crosslinks and/or complex-formation. Therefore we can conclude that dimers, multimers, or otherwise modified or cross-linked PLIN proteins might exist. Bulankina et al. [41] reported on the TIP47 migration into a high molecular weight band indicating the presence of dimers or trimers. In preliminary crosslink experiments using whole PLC cell material we found specific shifts and positive signals of higher molecular weight in immunoblots for adipophilin and TIP47 (not shown). We are currently trying with those experiments to identify additional binding partners.

The proteins MLDP and S3-12, detected in immunofluorescence microscopy as a very faint punctuate pattern, and found in enriched LD fractions by Western blotting were not found among the identified proteins within the proteomic analysis. Wolins et al. [42] reported that OA induced S3-12 and redistributed the protein in an insulin and glucose dependent manner. In our case under different conditions (short-time treatment of OA 3 h), we obviously could not induce S3-12 and therefore did not find any LDs with S3-12 in high buoyancy fractions. These data were confirmed by immunofluorescence microscopy where sizes of the LDs positive for MLDP and S3-12 appeared more or less unchanged in cells after short time OA treatment. However we could detect S3-12 by Western blotting within higher density gradient fractions, indicative of binding to very small LDs (LD2; **Fig. 4c**).

After analyzing the proteomic results, we decided to control purity of LD fractions by electron microscopy (EM) retrospectively. These EM controls of our LD1- and sLD- gradient fractions showed many relatively large LDs (one to several µm in diameter), but also many contaminants, inclusions of cytoplasm and general debris, even in the case where LDs had been intensively washed (“sLD”). Thus we had to concede, that even with the improved procedure, the result gave us enriched, but not pure LDs. These problems with “contaminations” in proteomic studies were also discussed by Fujimoto and Parton [43]. As such not all proteomic hits derived from such LD top layer preparations had something to do with LDs and, in contrary and importantly, some LD-binding proteins, especially those binding to presumably smaller LDs, did not show up in the top layer fraction. Those LD-binding proteins which do not appear in the commonly used top layer gradient fraction could therefore easily be overseen.

### LDs in Different Density Gradient Fractions

We switched our strategy and analyzed complete gradient separations. By immunoblotting, we found adipophilin essentially within three different gradient areas (albeit in different amounts; LD1, LD2 and LD3; see **Figs. 3** and **4c**). Importantly, S3-12 could be detected only in a higher density gradient fraction and not within the top layer. The finding of S3-12 in other than top layer gradient fractions was in agreement with the observation in immunofluorescence microscopy that S3-12 antibodies stained generally much smaller LDs when compared with adipophilin and TIP47 antibody staining [42]. These tiny and medium-sized LDs might have been missed in many studies solely working with ascending top layer fractions largely dealing with large fused LDs. Thus, Suzuki [44] reflected on the importance of the sizes of LDs, of lipid-protein ratios, and the authors emphasized the need for future work with very small LDs. Not the large-sized, fully developed or fused LDs from the gradient top layers, but small ones originating from early stages of LD biogenesis or endocytosis were assumed to be important. Information on very small LDs, however is lacking in current proteomic studies. We considered gradient fractions with higher densities being a better source for small LDs than the top layer fractions. We thought that our newly generated antibodies could address the major problems encountered with isolation and separation of these small LDs by density gradient centrifugation, i.e. co-migration with other cell components and organelles (ER, Golgi, PM complexes etc.). In our protocols, we used density gradient separation in conjunction with immunoprecipitations (IPs). We successfully precipitated from high density fractions our target proteins TIP47 and adipophilin and subsequently confirmed these proteins by Western blotting (not shown) and by mass spectrometry (MS) analysis (**Fig. 5**).

Most surprisingingly and strikingingly, we identified PLIN proteins in all fractions analyzed (LD1, LD2 and LD3) and co-precipitated intermediate-sized filament (IF) proteins, cytokeratin 8 and cytokeratin 18 with very high scores. Why cytokeratins were not found and/or did attract attention before in LD proteomic studies? Usually cytokeratins are deleted in proteomic studies as “finger” and “dandruff” contamination (for the “finger protein” cytokeratin 9 see [45]). Whereas this is probably hold true for cytokeratins derived from the epidermis - especially cytokeratins 1, 5, 9, 10 and 14 - this does not apply for cytokeratins 8 and 18. These cytokeratins are intrinsic proteins of the hepatocellular epithelium-derived PLC cells and definitely do not represent contaminations. Like in many cultured epithelial cells co-expression of vimentin is found (see **Fig. S6**; [46]). Umlauf and coworkers [47], working with epithelial cells of amniotic origin, already reported on LDs and cytokeratins 8 and 18 in addition to adipophilin and TIP47 and presented a list with many other polypeptides. However, these authors did not comment and did no further experiments involving these intermediate filaments. As many others authors working in the field, they were obviously biased in favor of LDs binding to tubulin as the only possibility. We found here no tubulin filaments in close contact to LDs with our EM immunolocalization studies. We have no confirmation on these proteins with a second independent method; especially with EM localization (see also discussion on EM data below). Therefore we did not consider the detected tubulin and actin hits by proteomics as members of potential direct LD-associated candidates. Studying LDs by arresting the tubulin system of cells (e.g. by nocodazole) also influences many other pathways, at least indirectly also the IF system (see, e.g., Lieber & Evans, [26]), Leung et al., [7]).

In addition, AUP1 homolog, known as a *bona fide* LD binding protein [48], [49], could be detected as co-precipitated protein with different antibodies against TIP47 and adipophilin (**Fig. 5**). Interestingly, we only identified this co-precipitation in higher density gradient fractions (LD3) and not in the top layer fractions (LD1). AUP1 has been reported to reside on LDs and seems to be involved in linking LDs via other proteins to ER protein, and is involved in quality controlling and the ubiquitination processes. Larson et al. [50], using rat insulinoma derived cells, and Sato et al. [51] with human hepatoma HepG2 cells also detected AUP1 with proteomic analysis in LD fractions.

Clathrin and AP2 adaptor protein were other co-precipitated proteins which could be identified (here again, as with AUP1 and other examples, not within the top layer gradient fraction LD1, but in fractions LD2 and LD3; **Fig. 5**). Both proteins are known to be targeted to specific receptor molecules and to play a central role in clathrin-mediated endocytosis and vesicles transport [52]–[54] (for the involvement of clathrin in mammalian cells in the uptake of hydrophobic components see also the LDL-receptor mediated pathway of plasma LDL and cholesterol reviewed by Brown and Goldstein [55]). Using OA treated Chinese hamster ovarian K2 cells, Bartz et al. [56] and Zehmer et al. [57] also reported the presence of clathrin and adaptin in LD proteomic studies. OA addition to the cell media seems to enhance clathrin-mediated endocytosis and, from our IPs, we conclude that TIP47 seems to be close to the coated pits and inasmuch involved.

Using antibodies to TIP47 and MLDP for IPs, we found one candidate termed “POTE ankyrin domain family member” within the gradient fractions LD2. Searching for ankyrin repeats in protein data bases, we discovered ankyrin-like repeat domains within the amino acid (aa) sequence of S3-12 (see **Figs. S8, S9**). Ankyrins are described to mediate protein-protein interactions. Interestingly, ankyrins were also characterized as intermediate filament acceptors [58].

### PLIN –Cytokeratin Double Immunofluorescence Microscopy

Within a dense meshwork of IFs and numerous LDs with OA treatment, it seems rather difficult to use the immunofluorescence microscopy method for demonstration of further PLIN-IF linkage, since the resolution of this method does not allow a definite conclusion on a direct co-localization (for resolution arguments see also [43]). However, we did get some localization data pointing to a specific relationship of PLIN and IF proteins. Upon higher magnification, using FA fixation and saponin treatment, we could detect many LDs indicative for possible co-localization of IF with PLIN antibody staining (**Figs. 7** **,** **8**). Especially when we used TIP47 antibodies, we saw small LDs sitting directly upon linear arrays of a filament system. This “docking” of small LDs alongside a filament system seemed to accumulate in the perinuclear area (**Figs. 8** and **S5**).

The OA uptake mechanism resembled in some aspects the Wolins et al. model [59], where the sequential transport of PLIN proteins from PM to perilipin-coated storage droplets - without describing the involvement of IFs - was investigated in adipocytes. These authors termed this special endocytotic process “LD maturation” and “model for LD biogenesis”. Actually, the LD biogenesis process seen in certain cells does not start with endocytotic processes but with “*de novo*” synthesis of LDs – as seen for example with the new expression of perilipin at the ER in adipocytes after specific stimulation, or the new expression of adipophilin and TIP47 in lactating mammary gland cells - thereby exhibiting droplets with phospholipid monolayer membranes. Therefore, those LDs derived from special receptor-mediated endocytosis should not be mixed up with the newly synthesized LDs inside of cells. In our opinion, the two routes leading to LDs in cells are completely different. Only in later processes these two types of LDs, the exogenously derived LDs (**exoLDs**) and the endogenously derived LDs (**endoLDs**) might fuse to larger LDs by a yet unknown mechanism. For formation of endoLDs, we favour the “membrane stalk” model of Fujimoto & Parton [43] as a model and possible explanation.

Upon OA stimulation, we noticed in addition to strong LD staining with antibodies for adipophilin, in some cases, a weak plasma membrane staining (e.g. **Fig. 7b,d**). These findings were in agreement with results obtained with freeze fractioning immunolocalization of adipophilin [**60**], [**61**] and with the reported plasma membrane source for isolation and localization of another PLIN family protein S3-12 [62].

In this current report, we explicitly used short-time OA stimulation (2–3 h) in order to obtain not too many and not too large LDs. With PLC cells we choose a cell line with a less dense network of cytokeratins when compared to many other epithelial cell lines. We thus intended to avoid a randomly created neighborship of IFs and LDs.

Both cell lines studied (liver carcinoma-derived PLC and the intestine carcinoma-derived CaCo-2 cells) express the IF proteins cytokeratins 8 and 18 and due to culturing conditions, also some vimentin as an additional IF protein (see [46]). But vimentin is definitely not expressed in all PLC cells and - compared to the cytokeratins - seems to be a minor component as judged by Western blots and immunofluorescence microscopy (**Fig. S6**). This expression, however, was sufficient that we could also detect vimentin by IP and MS (cp. **Figs. S4**).

Adipophilin could bind directly to TIP47 (e.g. see **Fig. 2a,b** **; 6c**) and upon OA treatment of cells, was found concentrated near or at the plasma membrane (**Figs. 7a,c**) and only later distributed throughout the cells, accumulating in a juxtanuclear region (examples shown in **Fig. 7e,f**). In this respect, the protein seemed to be involved in the trafficking of OA-containing droplets from the cell periphery to the perinuclear area. Usually adipophilin is seen bound to somewhat bigger-sized LDs in immunofluorescence microscopy when compared to TIP47 stained LDs. Now we found many situations with small LDs of TIP47 staining and small LDs of adipophilin staining which seemed just in the progress of combining and colocalization (see **Figs. 2a,b** **;** examples shown by arrowheads). Thus, with longer OA stimulation it could be that within larger sized, adipophilin-positively stained LDs - originating from fused TIP47 positive and adipophilin positive small LDs – the epitope for TIP47 is hidden and no longer accessible or detectable – and therefore only the adipophilin staining prevails in these large LDs.

MLDP and adipophilin too, showed co-localization in certain cells. Several LDs appeared with a mosaic-like mixed-colored immunofluorescence surface pattern, presumably indicating an uptake mechanism of hydrophobic components (see **Fig. 2d**).

As demonstrated with two different antibodies specific for TIP47 (**Fig. 2f** **)**, these antibodies might detect only certain pools of LDs within a given cell and do not necessarily show a complete overlap in staining and co-localization with all LDs present. Occassionally one of our N-terminal sequence derived PLIN antibodies did react somewhat differently from another antibody specific for the C-terminal sequence (not shown). Detailed studies on these phenomena are currently underway in our lab.

### Protein-Protein Interaction Assay

Based on our IP results, we further investigated a LD-PLIN-IF relationship. In protein-protein binding experiments on PVDF membranes, we could confirm that adipophilin, as well as TIP47, bind to cytokeratins and vice versa (**Fig. 6**). In addition, the binding experiments revealed a direct TIP47-adipophilin binding. This field of **PLIN-IF** interaction has to be complemented by additional specific crosslinking experiments in the future.

### LDs and EM Analysis

In our EM experiments we did see IF bundles directly bordering to LDs (**Fig. 9**) extending and confirming the evidence for such localizations obtained by IP experiments and protein-protein binding assays (**Figs. 5** **,** **6**). IFs comprise the largest gene family among the three major cytoskeleton protein groups in human and in many other organisms (for recent reviews see e.g. [63], [64]). No agenda to date lists IFs as essential partners within the context of LDs and only few authors (Suzuki et al. [44]) reason that LD distribution might not be controlled by microtubules alone; they emphasize that in adipocytes one finds IFs at the surface of LDs rather than microtubules, referring to the vimentin findings Franke and coworkers ([23] (see also references in the introduction). At the time of the Franke findings, the PLIN proteins were not yet discovered and therefore could not be incorporated in those studies. Our former immunoelectron microscopic work revealing IFs which could be seen in epithelial CaCo-2 cells in close proximity to LDs and adipophilin ([6]; see within this literature Fig. 8d) is of special interest in this context.

With all our EM data, we only recognized intermediate filaments close to LDs. As indicated in **Figure S4b-d**, tubulin was also identified with MS analysis in our IP experiments. Because tubulin filaments, in contrast to intermediate filament proteins, could not be confirmed in direct neighborhood of LDs by high resolution EM, we did not list or include this protein in **Figure 5**. We considered those proteins as not confirmed by a second independent method and therefore as an unspecific co-precipitation. In addition, neither mitochondria nor peroxisomes could be detected by EM directly linked to LDs so far. In all of our EM images there were always some interspace and distance or some ER leaflets found between these organelles. These observations might explain why LDs could be isolated and analysed without too much mitochondrial contamination. Evidence from many enzyme studies suggest, however, that there must be an exchange of lipophilic products between these two organelles, but at the moment our EM data speak against a direct and close membrane-to-membrane contact between LDs and mitochondria, at least in PLC cells.

Another important EM observation concerns the size of LDs. In our isolated LDs, obtained from gradient top layer fractions LD1 and sLD, an average diameter was estimated to be between 1–2 µm (see **Fig. S3**). The LDs connected with PLIN proteins and intermediate filaments in intact cells revealed much smaller droplets of 200–500 nm in diameters (see **Fig. 9**). Such differences again emphasize the need for new and different LD isolation methods. Commonly described isolation methods, use almost exclusively – independent of the gradient media used – the top layer gradient fractions for LDs. This focus on fast gradient ascending LDs and on large, probably fused, LDs, might not provide the true “original” LDs. Only in special cases and situations - like in adipocytes or in lipid disorders such as liver steatosis - might large LDs be formed in large quantities for easy recognition. The many, small LDs –the “normal LDs” - in tissues and cells are barely visible and may be easily overseen and as such not investigated. With the availability of antibodies to molecularly defined marker proteins, such as the LD-binding amphiphilic PLIN proteins, the situation should improve.

### Model for PLIN-IF Association

We analyzed the PLIN protein sequences with various programs of the Swiss Institute of Bioinformatics (SIB; see **Text S1**). Our question was: How can the PLIN proteins, without an obvious and known sequence domain, manage to bind specifically to hydrophobic substances and play such important roles in lipid transport and storage? Our speculation was, that the α-helices of the IF proteins might interact with the α-helical parts of the PLIN proteins. The X-ray structure of the C-terminal part of TIP47 showed four short α-helices (reported by Hickenbottom et al. ([65]). Our data base searches revealed alternating short hydrophobic/hydrophilic sequence patterns of PLIN proteins all along the sequences (**Fig. S7**). In addition, we found 29 ankyrin-like repeat units and one possible ACAT sequence motif for S3-12 (**Figs**. **S8** **,S9**).

Taken the above data together we suggest a model for possible **LD–PLIN** bindings and for **LD–PLIN–IF** interactions. Our considerations are schematically presented in **Figure 10**. This **LD–PLIN model** would not require specific amino acid domains for LD-binding (**Fig. 10a**). The many hydrophobic interactions by the many small hydrophobic units within the PLIN proteins could result in a strong binding of the protein to the LDs. In cases where parts of PLIN proteins are deleted or modified, e.g. by truncation or phosphorylation, the modified protein might lose it ability to bind strongly to LDs and - depending on the size of depletion or modification - might be released into the cytoplasma or replaced by other LD-binding proteins.

In summary we conclude: **(1)** The amphiphilic PLIN proteins are direct linkers of LDs and IFs. **(2)** Interaction of PLIN and IF proteins could be based on assemblies via α-helices. **(3)** S3-12 contains ankyrin-like repeats and a possible ACAT domain. **(4)** Future studies should concentrate on small LDs as described in our study.

## Supporting Information

Figure S1 **Characterization of generated primary mono- and polyclonal antibodies specific for human and mouse PLIN proteins by immunoblotting.** Whole cell lysates obtained from PLC cells (P, Coomassie blue stained lane in upper part) and from human adipose tissue (F, lane in lower part) were separated using gels without sample combs (“curtain gel”) by SDS-PAGE. After transfer to PVDF membranes and blocking with 0.2% Tween in PBS, antibodies were examined in a multi-slot apparatus (Biometra). Lanes 1,2: Different sera specific for the N-terminal peptide of adipophilin (pab Adipo-hNT-I 1-16; pab Adipo-hNT-II 6-27). Lanes 3,4: Different sera specific for the C-terminal peptide of adipophilin (pab Adipo-hCT). Lane 5: Sera specific for the N-terminal peptide of TIP47 (pab TIP47-hNT). Lanes 6,7: Different sera specific for a C-terminal peptide of TIP47 (pab TIP47-hCT). Lane 8: Sera specific for the C-terminal peptide of S3-12 (pab S3-12-hCT). Lane 9: Sera specific for Prp19p (This protein was described as LD-specific marker. Our sera stained exclusively nuclei and not LDs; [cp. Text S1 and literature SL1,2]). Lane 10: Sera specific for the N-terminal peptide of perilipin (pab Peri-h+mNT). Lanes 11,12: Different sera specific for the C-terminal peptide of perilipin (pab Peri-hCT). Note, whereas all PLIN antibodies - except those specific for perilipin - showed positive reactions with PLC cells, these antibodies were all completely negative with human fat cells. Perilipin sera showed strong positive reaction with fat, but not with PLC cells.
(TIF)

Figure S2 **Proteomic analysis of salt-washed gradient fraction sLD.** Complete gel lane shown in Fig. 4B was used for mass spectrometry analysis. Explanations on sample numbers, data base accession numbers of identified human proteins, color codes with preliminary assignments, brief protein descriptions, scores, predicted molecular weights, number of hits and other information are given at the top of the listing. Note: More than 650 proteins were identified. The blue color code is highlighting known LD-binding proteins. PLIN proteins adipophilin and TIP47 were detected in samples 7 and 8 of expected molecular weight with very high scores but these proteins could also be detected in samples of higher molecular weights. In sample numbers 7 and 8, Cytokeratins 8 and 18 were also identified with very high scores. Proteins involved in fatty acid, steroid- and lipid pathways were marked in red color code. Note in addition: Many of the given proteins were assigned by data base numbers only or could not be assigned exactly with the given information obtained from data bases. Therefore many of these assignments are preliminary and not confirmed.
(DOCX)

Figure S3 **Electron microscopic (EM) examination of density top layer fractions LD1 and sLD.** (**a**): Survey of fraction LD1; (**b**): Salt-washed fraction sLD; (cp. Figs. 3,4). Note: EM controls as purity control for isolated LDs have not been shown in LD proteomic studies so far. Even the salt-washed and re-centrifuged LD enriched fraction sLD (**b**) contained many contaminants, cytoplasm inclusions, membranous debris. By inspection of several such images, the average size of LDs of such preparations was found to have sizes of 1–2 µm in diameters. Bars: 5 µm.
(TIF)

Figure S4 **Proteomic analysis of immunoprecipitated density gradient fractions.** Fig. S4a: Designation of separated SDS-gel bands obtained from density gradients and specific immunoprecipitations (IPs) of OA stimulated PLC cells. Aliquots of each of the three gradient fractions (LD1, LD2 and LD3; cp. **Figs. 4c** **and** **5**) were used for IPs with monoclonal antibodies TIP47.49.12, MLDP 382.38 and AP125 (adipophilin). The used prefixes for analyzed silver-stained IP bands were numbered in the following way: T for TIP47 (T1–T13); M for MLDP (M1–M12) and A for adipophilin (A1–A12). Because we could not detect visible specific bands precipitated with the control antibody (VE-cadherin; see **Fig. 5**), we did not include those gel lanes for MS analysis. At the left margin the positions of molecular weight markers are given; at the right side position of co-precipitated background bands, i.e. immuoglobulins (IgG; heavy and light chains) and serum albumin (SA; derived from the fetal calf serum of hybridoma media). **Fig. S4b:** List of MS results obtained with mab for TIP47. **Fig. S4c:** List of identified proteins obtained with mab for MLDP. **Fig. S4d:** MS results of proteins obtained with mab AP125. Within the given lists are sample numbers, accession numbers, short protein descriptions, scores, molecular weights of identified protein and number of identified polypeptides. All identified IgGs, serum albumin, epidermal keratins and hits with very low scores were excluded. Color code used: yellow = PLIN proteins; blue = intermediate filament (IF) proteins; brown = AUP1 homolog protein. Note: Identified important proteins from these lists were already highlighted in **Figure 5**. (We did not include vimentin in **Fig. 5** (cp. **Fig. S6**; see also Discussion). Also not included in **Fig. 5** were the identified filament proteins actin and tubulin; in contrast to IFs, we could not confirm the localization of these proteins in EM close to LDs (see also Discussion).
(DOCX)

Figure S5 **Series of immunofluorescence microscopy images showing association of TIP47 with the IF network during OA uptake.** (**a–l**) Pattern variations of pab TIP47-hNT (red) of 3 h OA stimulated PLC cells are shown. (**a–c**) Within most cells a high number of small LDs could be seen. (**d–i**) Faint filamentous-like structures are regionally visible within the cytoplasm. (**h**) Upon closer look numerous tiny LDs could be detected sitting directly on filamentous structures. Dense rows of small droplets were obviously attached to a filament system. (**i–l**) In addition, with uptake of OA, some cells showed large LDs. (**i–k**) Some of the large LDs were found more at the cell periphery. (**l**) Others, bigger LDs, were distributed with ring-like appearance all over the cytoplasm. Fixation of the cells was with 2% formaldehyde/saponin; nuclear staining was with DAPI (blue). Bars: 10 µm.
(TIF)

Figure S6 **Detection of cytokeratins and of vimentin in PLC cells.** (**a–c′**) Western blot reactions of whole cell lysate of PLC, CaCo-2 and SV-80 cells. (**a**) Coomassie blue (Coom) stained PVDF membrane. (**b**) Pab cytokeratin 8 showed strong reactions with a 54 kD band with the two epithelial-derived cells, but not with fibroblast cell line. (**c,c′**) Different exposures of ECL-reaction using mab specific for vimentin revealed strong reaction with a 56 kD band in SV-80 cells. (**c′**) Only after longer exposure times an additional band was visible within the PLC lane (arrow). Position of molecular weight markers are indicated on the left margin. (**e–h**) Laser scanning double–label immunofluorescence microscopy showing comparisons of cytokeratin and vimentin staining. (**e,f**) Mab specific for cytokeratin 8 (red) was incubated together with pab specific for vimentin (green). (**g,h**) A different combination of antibodies was used: mab for vimentin (red) and pab for cytokeratin 8 (green). Note: In contrast to the cytokeratin staining which showed a strong IF staining in all cells, the vimentin staining (using two different antibodies) is not seen in all cells and preferentially only locally near the cell nucleus. We conclude that within those PLC cells - negative for vimentin - cytokeratins 8 and 18 are the only IF protein candidates for LD binding. Bars: 10 µm.
(TIF)

Figure S7 **Hydrophobicity analysis of adipophilin and multiple sequence alignment (MSA) of adipophilin, TIP47 and MLDP. Fig. S7a:** Adipophilin showed many alternating hydrophilic/hydrophobic short stretches of amino acids. This general pattern, seen overall within the primary protein sequences of PLIN family members, was generated using “Windows size 3” with the Kyte/Doolittle program of ProtScale and the EXPASY server of Swiss Institute of Bioinformatics (SIB) [see Text S1; SL3-6]. **Fig. S7b:** PLIN sequences were run with the TCoffee and MSA hub programs (SIB). The result could be viewed with Jalview 2 Launcher and the “hydrophobicity colour mode” [for jalview program see Text S1; SL7]. Note: The many alternating hydrophobic/hydrophilic sequence mini-domains all over the complete sequences. These alternate changes might be the major reason for the amphiphilic properties of the PLIN proteins, leading us to propose a model for LD-PLIN protein binding (see **Fig. 10a**).
(TIF)

Figure S8 **Sequence alignment of the human PLIN protein family, α-helices and ankyrin repeats.** MSA was performed with the program “ClustalW” at Expasy (SIB). Sequence positions with identical amino acids within the alignment were colored equally and are reflecting the homology of the protein family. The four α-helical domains at the C-terminus of adipophilin, TIP47, MLDP and S3-12 were boxed. Perilipin possessed no α-helices, but an E-rich domain (boxed; green letters). 29 ankyrin-like repeats were found at the N-terminal and central region of S3-12 (highlighted alternatively in red and purple).
(TIF)

Figure S9 **Multiple sequence alignment (MSA) of the repeat units of S3-12.** Ankyrin-like repeats, previously unnoticed, were found at the N-terminal sequence and in the central part of the S3-12 sequence. These repeats contain the classical number of 33 amino acids for ankyrins and 2 helical segments within each repeat, i.e. the repeats exhibit modules with helix-turn helix conformation (Bottom, right side; [modified from literature SL8,9 in Text S1]). Each ankyrin-like repeat sequence was separately applied, as single 33 aa peptide sequence, in computer MSA programs. Alignment runs were with the TCoffee programs of SIB for multiple alignments. The MSA result was viewed with Jalview 2 Launcher [for jalview program see Text S1; SL7]. The alignments shown are visualized by Clustal X, by Hydrophobicity and by Helix Propensity (with highlighted cysteins in yellow) applied colour programs. Note: The image obtained with the hydrophobicity modus showed in this C-terminal and central part of S3-12 many alternating hydrophilic (blue)/hydrophobic (red) short aa domains. These characteristics were already shown with other PLIN proteins (Fig. S7) and might give S3-12 additional capacities to display amphiphilic protein properties by potential similar folding and binding to LDs as shown in our model of **Fig. 10A** **.**
(TIF)

Table S1 **Summary list of the new antibodies generated against lipid droplet (LD) -binding proteins.** Antibody designation, animal source and amino acid (aa) positions of used peptides of PLIN proteins or used recombinant PLIN proteins for immunization are listed. Polypeptides were synthesized (PSL, Heidelberg, Germany) and conjugated to keyhole limpet hemocyanin (KLH) to trigger and enhance immunoreaction. NT = N-terminal; CT = C-terminal; h = human; m = mouse; gp = guinea pig; mab = monoclonal antibody; pab = polyclonal antibody; amino acid; (aa) = positions of peptides used; rec = human recombinant PLIN proteins used for generation of mabs. The full length clones of the human proteins TIP47 and MLDP were purchased from ImaGenes (Berlin, Germany; ORF Expression clone IOH6849 and I.M.A.G.E. cDNA clone IRCMp5012F0237D) and used for the production of recombinant proteins. With these proteins monoclonal antibodies were generated together with the Monoclonal Antibody Core Facility of the German Cancer Research Center (Heidelberg, Germany). Monoclonal antibodies specific for adipophilin and perilipin were generated in the Helmholtz Group for Cell Biology (German Cancer Research Center) using KLH-coupled polypeptides for immunization and BALB/c mice. Most of the listed antibodies are commercially available from PROGEN Biotechnik, Heidelberg.
(DOCX)

Text S1 **Remarks on ankyrin-like repeats, data base searches and literature SL1–SL13 for Supporting Information.**
(DOCX)
