# Supplementary figures and images for: Lipid Droplets, Perilipins and Cytokeratins – Unravelled Liaisons in Epithelium-Derived Cells
Source: PLoS One. 2013 May 21;8(5):e63061. doi: 10.1371/journal.pone.0063061 (PMC3660578; doi:10.1371/journal.pone.0063061)

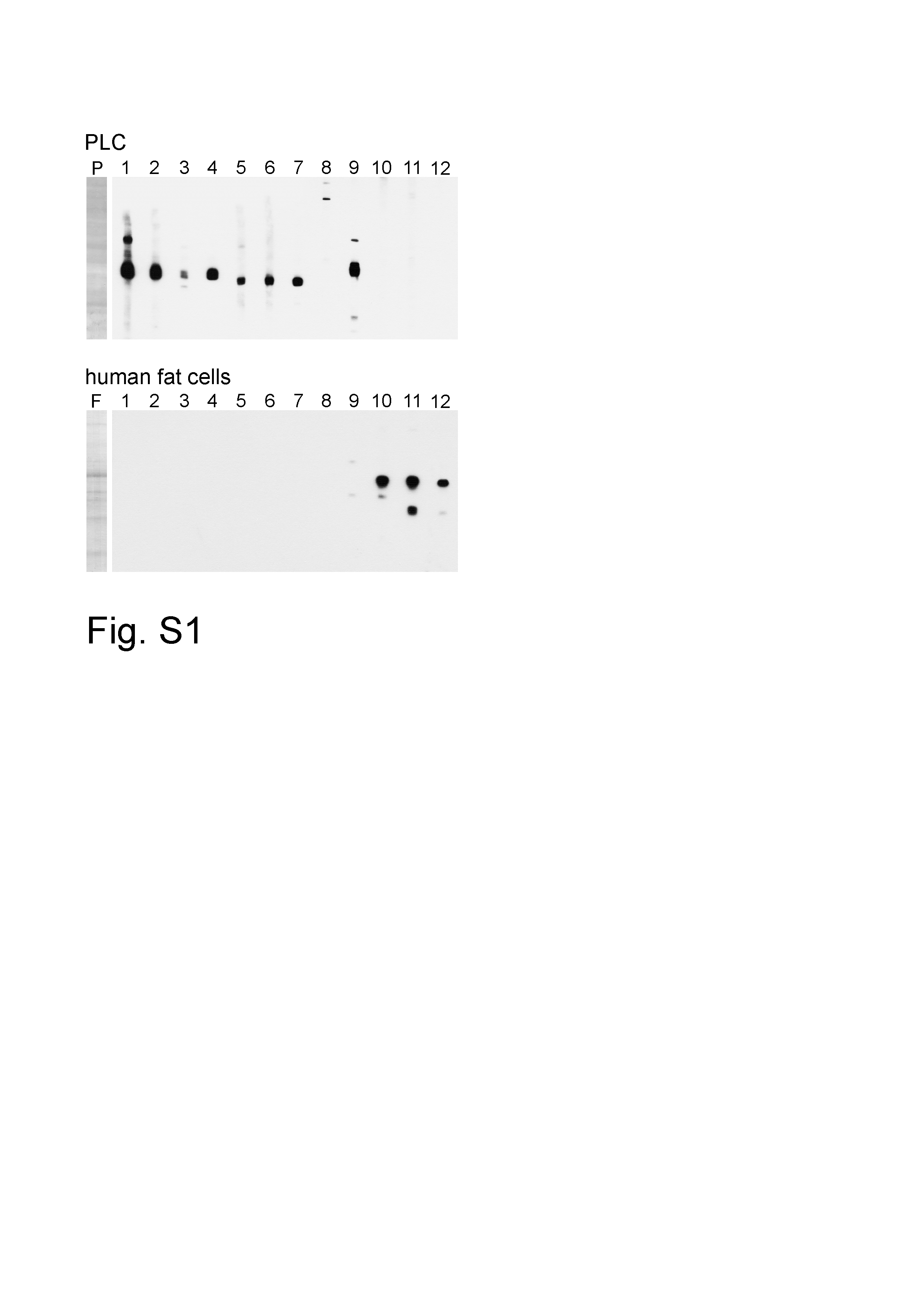

Supplement: Figure S1 — Characterization of generated primary mono- and polyclonal antibodies specific for human and mouse PLIN proteins by immunoblotting. Whole cell lysates obtained from PLC cells (P, Coomassie blue stained lane in upper part) and from human adipose tissue (F, lane in lower part) were separated using gels without sample combs (“curtain gel”) by SDS-PAGE. After transfer to PVDF membranes and blocking with 0.2% Tween in PBS, antibodies were examined in a multi-slot apparatus (Biometra). Lanes 1,2: Different sera specific for the N-terminal peptide of adipophilin (pab Adipo-hNT-I 1-16; pab Adipo-hNT-II 6-27). Lanes 3,4: Different sera specific for the C-terminal peptide of adipophilin (pab Adipo-hCT). Lane 5: Sera specific for the N-terminal peptide of TIP47 (pab TIP47-hNT). Lanes 6,7: Different sera specific for a C-terminal peptide of TIP47 (pab TIP47-hCT). Lane 8: Sera specific for the C-terminal peptide of S3-12 (pab S3-12-hCT). Lane 9: Sera specific for Prp19p (This protein was described as LD-specific marker. Our sera stained exclusively nuclei and not LDs; [cp. Text S1 and literature SL1,2]). Lane 10: Sera specific for the N-terminal peptide of perilipin (pab Peri-h+mNT). Lanes 11,12: Different sera specific for the C-terminal peptide of perilipin (pab Peri-hCT). Note, whereas all PLIN antibodies - except those specific for perilipin - showed positive reactions with PLC cells, these antibodies were all completely negative with human fat cells. Perilipin sera showed strong positive reaction with fat, but not with PLC cells. (TIF) [file pone.0063061.s001.tif]

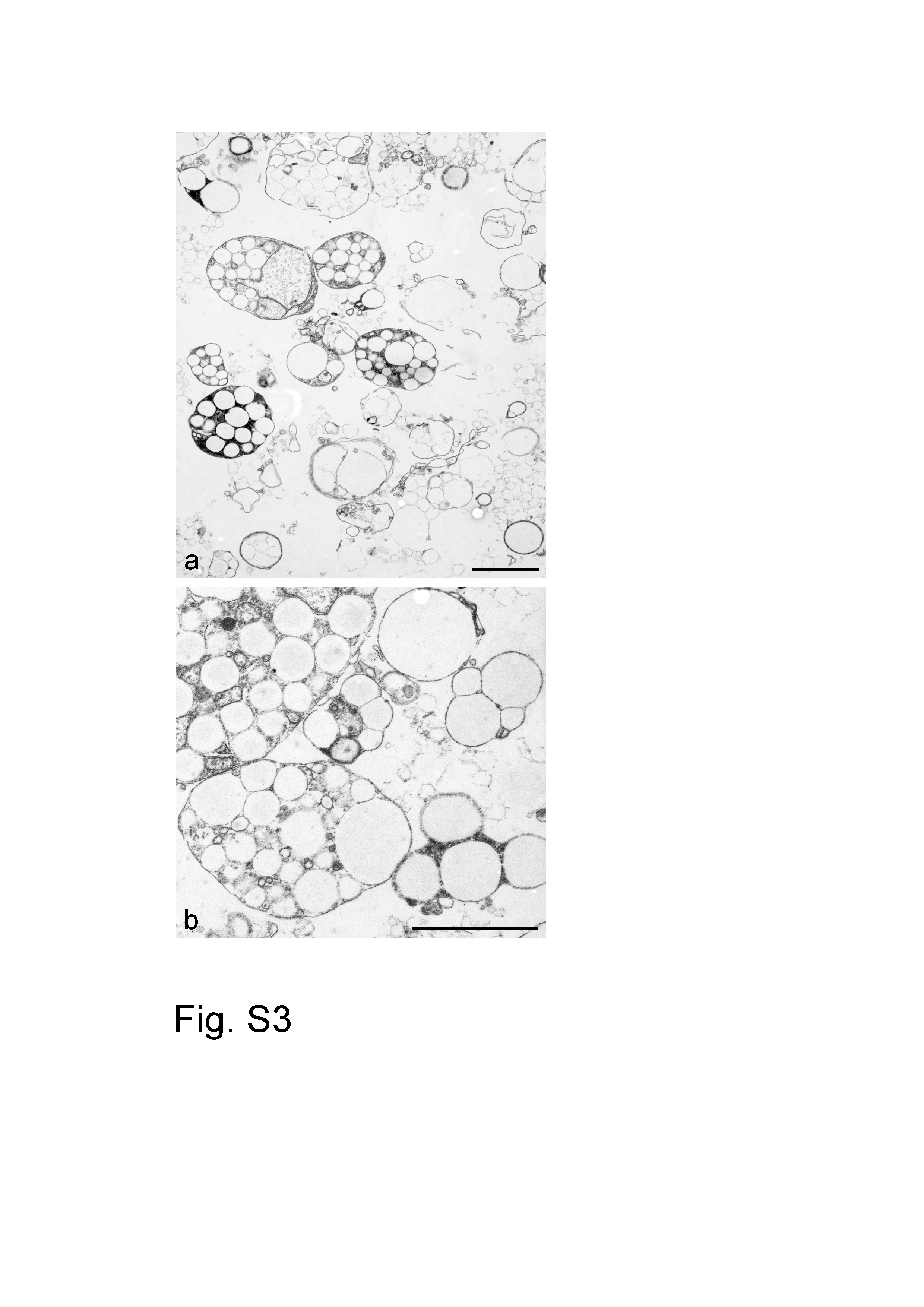

Supplement: Figure S3 — Electron microscopic (EM) examination of density top layer fractions LD1 and sLD. (a): Survey of fraction LD1; (b): Salt-washed fraction sLD; (cp. Figs. 3,4). Note: EM controls as purity control for isolated LDs have not been shown in LD proteomic studies so far. Even the salt-washed and re-centrifuged LD enriched fraction sLD (b) contained many contaminants, cytoplasm inclusions, membranous debris. By inspection of several such images, the average size of LDs of such preparations was found to have sizes of 1–2 µm in diameters. Bars: 5 µm. (TIF) [file pone.0063061.s003.tif]

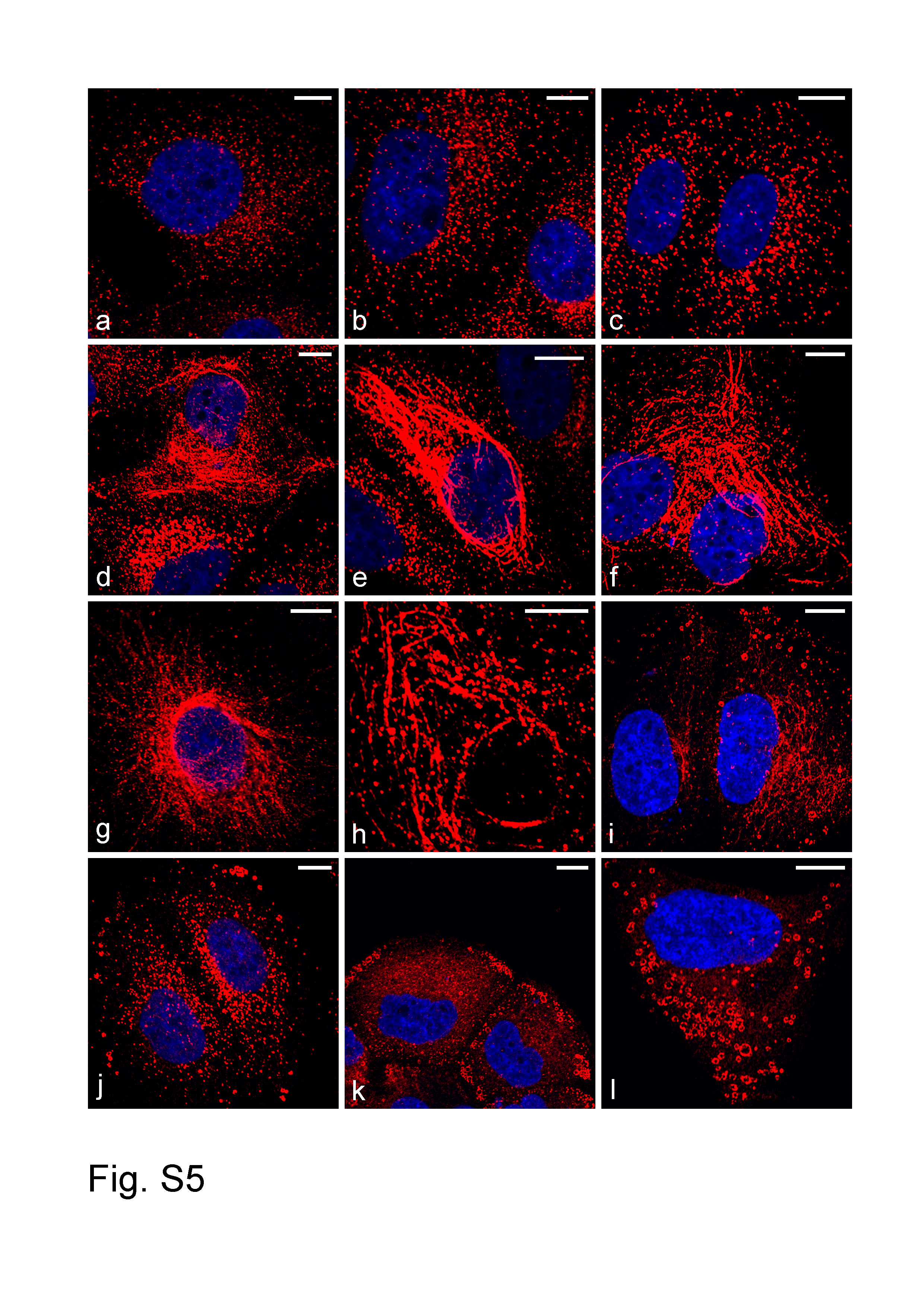

Supplement: Figure S5 — Series of immunofluorescence microscopy images showing association of TIP47 with the IF network during OA uptake. (a–l) Pattern variations of pab TIP47-hNT (red) of 3 h OA stimulated PLC cells are shown. (a–c) Within most cells a high number of small LDs could be seen. (d–i) Faint filamentous-like structures are regionally visible within the cytoplasm. (h) Upon closer look numerous tiny LDs could be detected sitting directly on filamentous structures. Dense rows of small droplets were obviously attached to a filament system. (i–l) In addition, with uptake of OA, some cells showed large LDs. (i–k) Some of the large LDs were found more at the cell periphery. (l) Others, bigger LDs, were distributed with ring-like appearance all over the cytoplasm. Fixation of the cells was with 2% formaldehyde/saponin; nuclear staining was with DAPI (blue). Bars: 10 µm. (TIF) [file pone.0063061.s005.tif]

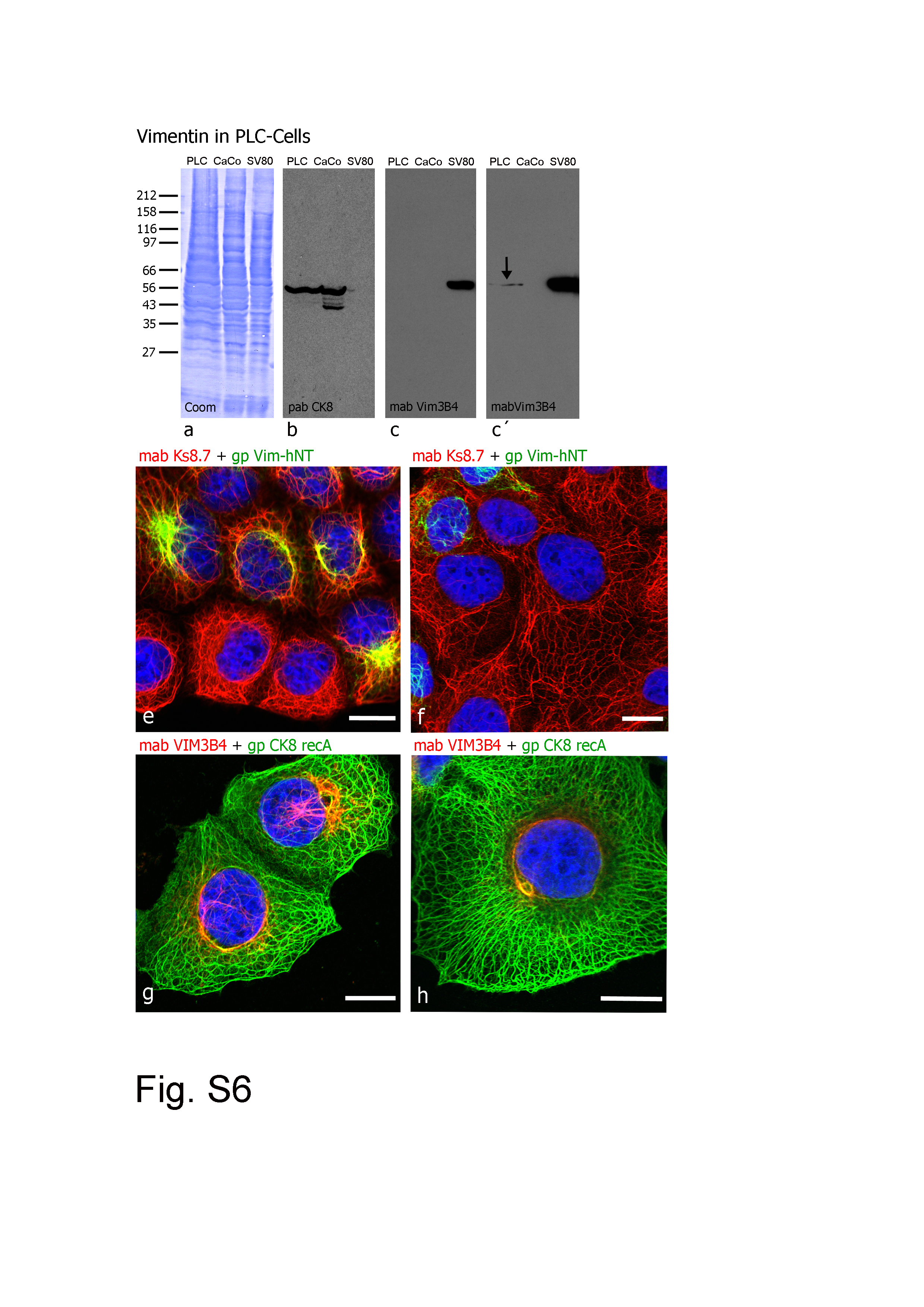

Supplement: Figure S6 — Detection of cytokeratins and of vimentin in PLC cells. (a–c′) Western blot reactions of whole cell lysate of PLC, CaCo-2 and SV-80 cells. (a) Coomassie blue (Coom) stained PVDF membrane. (b) Pab cytokeratin 8 showed strong reactions with a 54 kD band with the two epithelial-derived cells, but not with fibroblast cell line. (c,c′) Different exposures of ECL-reaction using mab specific for vimentin revealed strong reaction with a 56 kD band in SV-80 cells. (c′) Only after longer exposure times an additional band was visible within the PLC lane (arrow). Position of molecular weight markers are indicated on the left margin. (e–h) Laser scanning double–label immunofluorescence microscopy showing comparisons of cytokeratin and vimentin staining. (e,f) Mab specific for cytokeratin 8 (red) was incubated together with pab specific for vimentin (green). (g,h) A different combination of antibodies was used: mab for vimentin (red) and pab for cytokeratin 8 (green). Note: In contrast to the cytokeratin staining which showed a strong IF staining in all cells, the vimentin staining (using two different antibodies) is not seen in all cells and preferentially only locally near the cell nucleus. We conclude that within those PLC cells - negative for vimentin - cytokeratins 8 and 18 are the only IF protein candidates for LD binding. Bars: 10 µm. (TIF) [file pone.0063061.s006.tif]

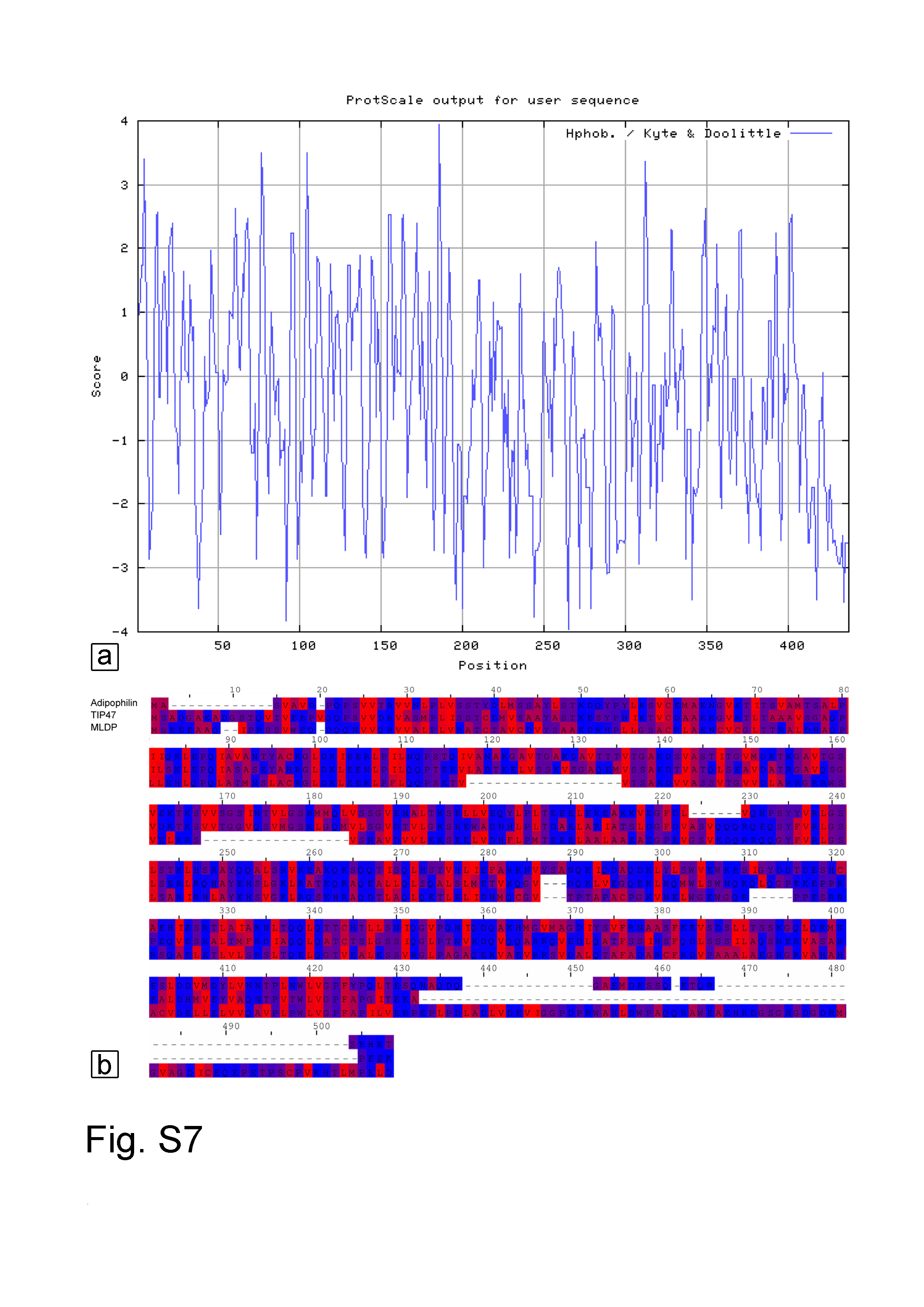

Supplement: Figure S7 — Hydrophobicity analysis of adipophilin and multiple sequence alignment (MSA) of adipophilin, TIP47 and MLDP. Fig. S7a: Adipophilin showed many alternating hydrophilic/hydrophobic short stretches of amino acids. This general pattern, seen overall within the primary protein sequences of PLIN family members, was generated using “Windows size 3” with the Kyte/Doolittle program of ProtScale and the EXPASY server of Swiss Institute of Bioinformatics (SIB) [see Text S1; SL3-6]. Fig. S7b: PLIN sequences were run with the TCoffee and MSA hub programs (SIB). The result could be viewed with Jalview 2 Launcher and the “hydrophobicity colour mode” [for jalview program see Text S1; SL7]. Note: The many alternating hydrophobic/hydrophilic sequence mini-domains all over the complete sequences. These alternate changes might be the major reason for the amphiphilic properties of the PLIN proteins, leading us to propose a model for LD-PLIN protein binding (see Fig. 10a ). (TIF) [file pone.0063061.s007.tif]

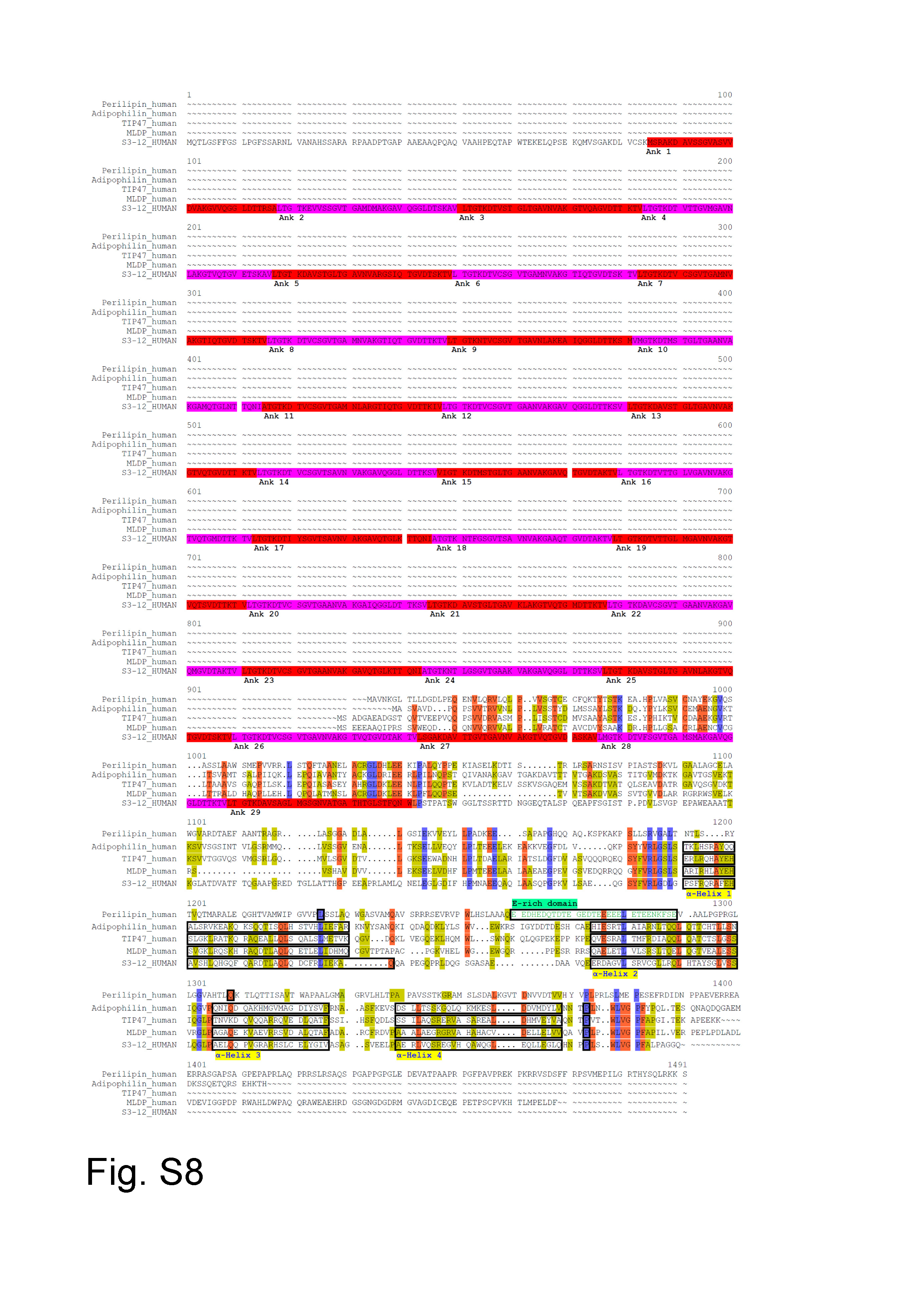

Supplement: Figure S8 — Sequence alignment of the human PLIN protein family, α-helices and ankyrin repeats. MSA was performed with the program “ClustalW” at Expasy (SIB). Sequence positions with identical amino acids within the alignment were colored equally and are reflecting the homology of the protein family. The four α-helical domains at the C-terminus of adipophilin, TIP47, MLDP and S3-12 were boxed. Perilipin possessed no α-helices, but an E-rich domain (boxed; green letters). 29 ankyrin-like repeats were found at the N-terminal and central region of S3-12 (highlighted alternatively in red and purple). (TIF) [file pone.0063061.s008.tif]

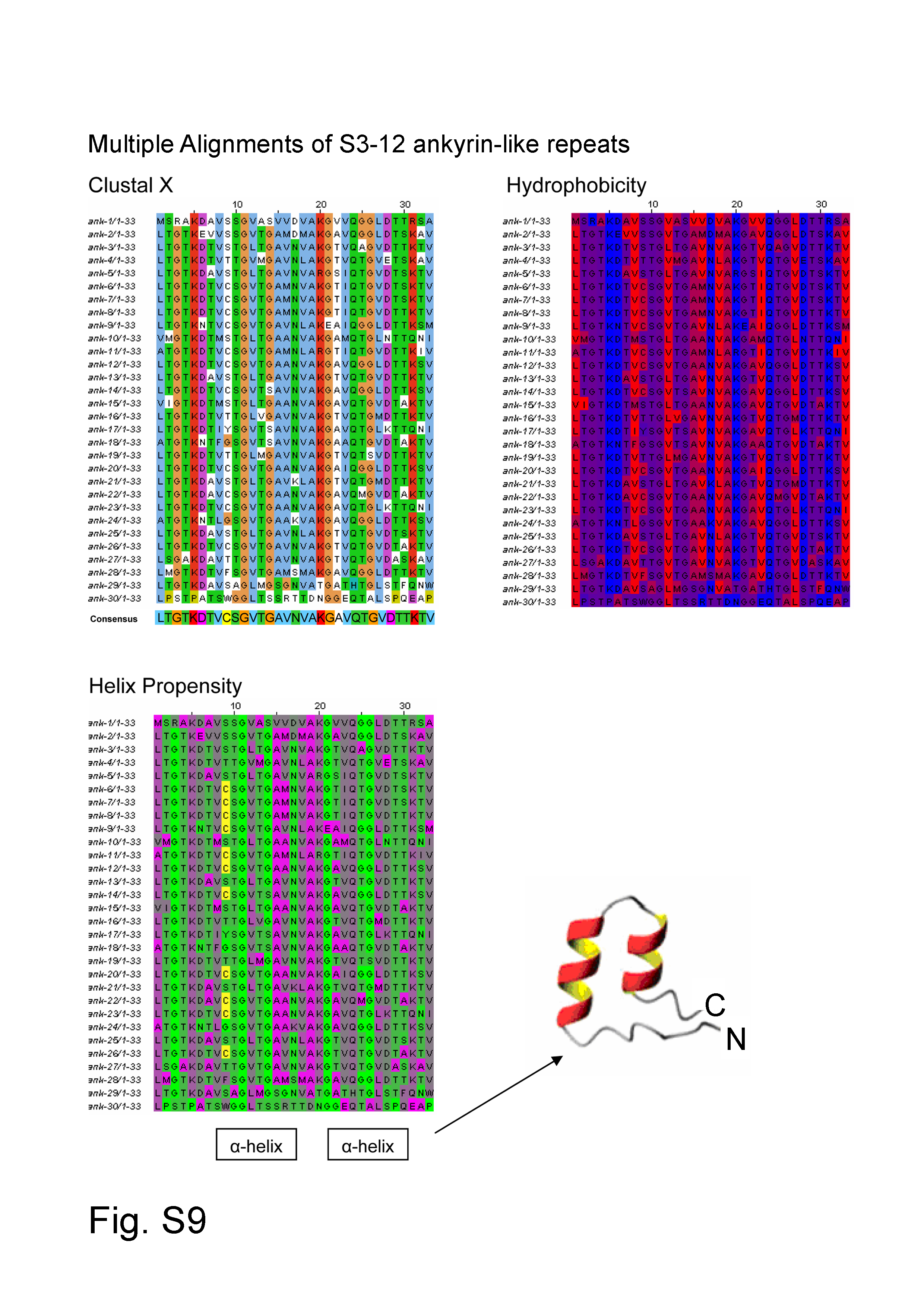

Supplement: Figure S9 — Multiple sequence alignment (MSA) of the repeat units of S3-12. Ankyrin-like repeats, previously unnoticed, were found at the N-terminal sequence and in the central part of the S3-12 sequence. These repeats contain the classical number of 33 amino acids for ankyrins and 2 helical segments within each repeat, i.e. the repeats exhibit modules with helix-turn helix conformation (Bottom, right side; [modified from literature SL8,9 in Text S1]). Each ankyrin-like repeat sequence was separately applied, as single 33 aa peptide sequence, in computer MSA programs. Alignment runs were with the TCoffee programs of SIB for multiple alignments. The MSA result was viewed with Jalview 2 Launcher [for jalview program see Text S1; SL7]. The alignments shown are visualized by Clustal X, by Hydrophobicity and by Helix Propensity (with highlighted cysteins in yellow) applied colour programs. Note: The image obtained with the hydrophobicity modus showed in this C-terminal and central part of S3-12 many alternating hydrophilic (blue)/hydrophobic (red) short aa domains. These characteristics were already shown with other PLIN proteins (Fig. S7) and might give S3-12 additional capacities to display amphiphilic protein properties by potential similar folding and binding to LDs as shown in our model of Fig. 10A . (TIF) [file pone.0063061.s009.tif]
