# Supplementary material for: Lipid Droplets, Perilipins and Cytokeratins – Unravelled Liaisons in Epithelium-Derived Cells
Source: PLoS One. 2013 May 21;8(5):e63061. doi: 10.1371/journal.pone.0063061 (PMC3660578; doi:10.1371/journal.pone.0063061)
Supplement: Figure S2 — Proteomic analysis of salt-washed gradient fraction sLD. Complete gel lane shown in Fig. 4B was used for mass spectrometry analysis. Explanations on sample numbers, data base accession numbers of identified human proteins, color codes with preliminary assignments, brief protein descriptions, scores, predicted molecular weights, number of hits and other information are given at the top of the listing. Note: More than 650 proteins were identified. The blue color code is highlighting known LD-binding proteins. PLIN proteins adipophilin and TIP47 were detected in samples 7 and 8 of expected molecular weight with very high scores but these proteins could also be detected in samples of higher molecular weights. In sample numbers 7 and 8, Cytokeratins 8 and 18 were also identified with very high scores. Proteins involved in fatty acid, steroid- and lipid pathways were marked in red color code. Note in addition: Many of the given proteins were assigned by data base numbers only or could not be assigned exactly with the given information obtained from data bases. Therefore many of these assignments are preliminary and not confirmed. (DOCX) [file pone.0063061.s002.docx]

Fig. S2
