# Supplementary material for: Lipid Droplets, Perilipins and Cytokeratins – Unravelled Liaisons in Epithelium-Derived Cells
Source: PLoS One. 2013 May 21;8(5):e63061. doi: 10.1371/journal.pone.0063061 (PMC3660578; doi:10.1371/journal.pone.0063061)
Supplement: Figure S4 — Proteomic analysis of immunoprecipitated density gradient fractions. Fig. S4a: Designation of separated SDS-gel bands obtained from density gradients and specific immunoprecipitations (IPs) of OA stimulated PLC cells. Aliquots of each of the three gradient fractions (LD1, LD2 and LD3; cp. Figs. 4c and 5 ) were used for IPs with monoclonal antibodies TIP47.49.12, MLDP 382.38 and AP125 (adipophilin). The used prefixes for analyzed silver-stained IP bands were numbered in the following way: T for TIP47 (T1–T13); M for MLDP (M1–M12) and A for adipophilin (A1–A12). Because we could not detect visible specific bands precipitated with the control antibody (VE-cadherin; see Fig. 5 ), we did not include those gel lanes for MS analysis. At the left margin the positions of molecular weight markers are given; at the right side position of co-precipitated background bands, i.e. immuoglobulins (IgG; heavy and light chains) and serum albumin (SA; derived from the fetal calf serum of hybridoma media). Fig. S4b: List of MS results obtained with mab for TIP47. Fig. S4c: List of identified proteins obtained with mab for MLDP. Fig. S4d: MS results of proteins obtained with mab AP125. Within the given lists are sample numbers, accession numbers, short protein descriptions, scores, molecular weights of identified protein and number of identified polypeptides. All identified IgGs, serum albumin, epidermal keratins and hits with very low scores were excluded. Color code used: yellow = PLIN proteins; blue = intermediate filament (IF) proteins; brown = AUP1 homolog protein. Note: Identified important proteins from these lists were already highlighted in Figure 5 . (We did not include vimentin in Fig. 5 (cp. Fig. S6; see also Discussion). Also not included in Fig. 5 were the identified filament proteins actin and tubulin; in contrast to IFs, we could not confirm the localization of these proteins in EM close to LDs (see also Discussion). (DOCX) [file pone.0063061.s004.docx]

Fig. S4a

Fig. S4b

Fig. S4c

Fig. S4d
