# Supplementary material for: Lipid Droplets, Perilipins and Cytokeratins – Unravelled Liaisons in Epithelium-Derived Cells
Source: PLoS One. 2013 May 21;8(5):e63061. doi: 10.1371/journal.pone.0063061 (PMC3660578; doi:10.1371/journal.pone.0063061)
Supplement: Text S1 — Remarks on ankyrin-like repeats, data base searches and literature SL1–SL13 for Supporting Information. (DOCX) [file pone.0063061.s011.docx]

**Text S1:** *Remarks on ankyrin-like repeats, data base searches and literature SL1-SL13 for Supporting Information.* Ankyrins were found to be localized close to plasma membranes (PMs) and comprise a family playing a role for multiple membrane transporters, known as anchors of spectrin-based membrane skeleton [SL[10](#_ENREF_10)]. An original report on the cloning and sequencing of a PM protein named S3-12, mentioned 29 repeats and homology with adipophilin, but did not comment on these repeats as possible ankyrin members [SL[11](#_ENREF_11)]. We now found with sequence comparisons that these unusually high numbers of repeats could be previously unnoticed ankyrin-like repeats [for computer programs of the EXPASY server and “SUPERFAMILY” search see SL[5](#_ENREF_5)]. Additionally this program suggests one thiolase-like domain for S3-12, which is also described as an acetyl-Coenzyme A acetyltransferase (ACAT), a key enzyme in degradative pathways such as fatty acid beta-oxidation and the involvement in the synthesis of fatty acids and steroids (cp. also our findings of IPs and detection of ACAT with mab TIP47). Each repeat comprises 33 aa - the number of classical ankyrins - and exhibits modules with a helix-turn helix conformation (see **Fig. S7**). We further searched with other computer programs of the EXPASY server and found, e.g., with T-REKS not all, but 20 repeats of 33 aa length [SL[12](#_ENREF_12)]. But none of these other programs suggested a family relationship to ankyrins for S3-12. This aspect of ankyrin-like repeats within PLIN- proteins might be the link for the reported plasma membrane (PM) localization of PLIN protein antibodies [SL[11](#_ENREF_11), SL[13](#_ENREF_13)]. At the PM, together with the other identified IP proteins (clathrin, AP2-adaptor protein), in particular S3-12 might be capable and involved in the uptake of lipids and hydrophobic substances.

Literature for Supporting Information:

SL1. Cho SY, Park PJ, Lee JH, Kim JJ, Lee TR (2007) Identification of the domains required for the localization of Prp19p to lipid droplets or the nucleus. Biochem Biophys Res Commun 364: 844-849.

SL2. Cho SY, Shin ES, Park PJ, Shin DW, Chang HK, et al. (2007) Identification of mouse Prp19p as a lipid droplet-associated protein and its possible involvement in the biogenesis of lipid droplets. J Biol Chem 282: 2456-2465.

SL3. Gasteiger E, Jung E, Bairoch A (2001) SWISS-PROT: connecting biomolecular knowledge via a protein database. Curr Issues Mol Biol 3: 47-55.

SL4. Bairoch A, Apweiler R, Wu CH, Barker WC, Boeckmann B, et al. (2005) The Universal Protein Resource (UniProt). Nucleic Acids Res 33: D154-159.

SL5. Gough J, Karplus K, Hughey R, Chothia C (2001) Assignment of homology to genome sequences using a library of hidden Markov models that represent all proteins of known structure. J Mol Biol 313: 903-919.

SL6. Artimo P, Jonnalagedda M, Arnold K, Baratin D, Csardi G, et al. (2012) ExPASy: SIB bioinformatics resource portal. Nucleic Acids Res 40: W597-W603.

SL7. Waterhouse AM, Procter JB, Martin DM, Clamp M, Barton GJ (2009) Jalview Version 2--a multiple sequence alignment editor and analysis workbench. Bioinformatics 25: 1189-1191.

SL8. Li J, Mahajan A, Tsai MD (2006) Ankyrin repeat: a unique motif mediating protein-protein interactions. Biochemistry 45: 15168-15178.

SL9. Michaely P, Tomchick DR, Machius M, Anderson RG (2002) Crystal structure of a 12 ANK repeat stack from human ankyrinR. EMBO J 21: 6387-6396.

SL10. Bennett V, Stenbuck PJ (1979) Identification and partial purification of ankyrin, the high affinity membrane attachment site for human erythrocyte spectrin. J Biol Chem 254: 2533-2541.

SL11. Scherer PE, Bickel PE, Kotler M, Lodish HF (1998) Cloning of cell-specific secreted and surface proteins by subtractive antibody screening. Nat Biotechnol 16: 581-586.

SL12. Jorda J, Kajava AV (2009) T-REKS: identification of Tandem REpeats in sequences with a K-meanS based algorithm. Bioinformatics 25: 2632-2638.

SL13. Robenek H, Robenek MJ, Buers I, Lorkowski S, Hofnagel O, et al. (2005) Lipid droplets gain PAT family proteins by interaction with specialized plasma membrane domains. J Biol Chem 280: 26330-26338.
